# SOIL-WATERGRIDS, mapping dynamic changes in soil moisture and depth of water table from 1970 to 2014

**DOI:** 10.1038/s41597-021-01032-4

**Published:** 2021-10-06

**Authors:** Magda Guglielmo, Fiona H. M. Tang, Chiara Pasut, Federico Maggi

**Affiliations:** Laboratory for Advanced Environmental Engineering Research, School of Civil Engineering, The University of Sydney, Bld. J05, 2006 Sydney, NSW Australia

**Keywords:** Hydrology, Scientific data

## Abstract

We introduce here SOIL-WATERGRIDS, a new dataset of dynamic changes in soil moisture and depth of water table over 45 years from 1970 to 2014 globally resolved at 0.25 × 0.25 degree resolution (about 30 × 30 km at the equator) along a 56 m deep soil profile. SOIL-WATERGRIDS estimates were obtained using the BRTSim model instructed with globally gridded soil physical and hydraulic properties, land cover and use characteristics, and hydrometeorological variables to account for precipitation, ecosystem-specific evapotranspiration, snowmelt, surface runoff, and irrigation. We validate our estimates against independent observations and re-analyses of the soil moisture, water table depth, wetland occurrence, and runoff. SOIL-WATERGRIDS brings into a single product the monthly mean water saturation at three depths in the root zone and the depth of the highest and lowest water tables throughout the reference period, their long-term monthly averages, and data quality. SOIL-WATERGRIDS can therefore be used to analyse trends in water availability for agricultural abstraction, assess the water balance under historical weather patterns, and identify water stress in sensitive managed and unmanaged ecosystems.

## Abstract (summary)

**Table:** 

Measurement(s)	volumetric water content • water table depth
Technology Type(s)	computational modeling technique
Factor Type(s)	hydrometeorology • soil properties • land use
Sample Characteristic - Environment	soil

Machine-accessible metadata file describing the reported data: 10.6084/m9.figshare.15982578

## Background & Summary

Groundwater provides about 33% of total water withdrawals worldwide^1^; this fulfils about 35% of the global drinking water demand^2^ and about 40% of irrigation water in agricultural areas^3,4^. However, continued groundwater abstraction can decrease the storage and threaten ecosystems and communities that depend on below-surface water, especially in arid and semiarid regions^4–6^. For instance, near surface water tables can regulate the soil water content at the surface through capillary rise^7^, supply water to the root zone supporting plant transpiration, and alleviate soil evaporation not only in arid and semiarid regions^8–10^ but also where seasonal rainfalls alternate with long dry periods^11,12^. In addition to anthropogenic factors, climate variability is a concurrent threat to the groundwater availability because it can alter the long-term aquifer and vadose zone water budget and can influence, as a consequence, the making of groundwater use policies^13,14^. Knowing the state of groundwater globally is essential to identify where groundwater provides vital support to the ecosystems and their environmental services^15^. Near-surface soil water measurements are usually conducted through borehole sampling. Ideally, the greater the density of monitoring stations per surface area, the greater the data reliability and usability. However, the monitoring infrastructure is expensive and requires continuing maintenance and manual data logging in the majority of cases. Despite a large number of boreholes worldwide (more than 1.6 million^15^), the density per surface area is not enough to produce spatially uniform assessments of the actual groundwater storage over wide geographical regions.

Currently, several georeferenced gridded datasets are available for global water resources. The most extensive catalogue of the groundwater table depth is provided by^10^, which combines global observations from government archives and the literature with estimates by hydrological models. Although this dataset distributes the equilibrium (long-term mean) water table depth and its long-term monthly averages from 2004 to 2013, it does not provide information about the surface and root-zone soil water content, which is fundamentally dependent on the proximity to the water table. On the other hand, existing satellite data provide distributed records of the soil water content at different depths near-surface (generally within the top 30 cm^16,17^), while soil moisture sensor measurements provide recordings to deeper depths^18^ but their spatial coverage is often limited to more or less extended regions. Advanced land surface models combined with data assimilation techniques (e.g., GLDAS model in^19^) have contributed to expanding soil moisture data by filling in spatial and temporal gaps.

The datasets mentioned above provide invaluable information on the global water asset. However, the existing estimates of water table depth and near-surface soil moisture are independent from each other and independently validated. Also, these have a different level of detail (e.g., resolution, depth) and report different target variables (e.g., volumetric soil water content, soil water saturation, water table depth). Most importantly, no set of data currently exists for the time- and depth-resolved soil water content or saturation in the topsoil, root zone, and vadose zone and the water table depth (one or multiple) over the same geographic grid. The lack of knowledge of the water connectivity between near-surface and groundwater compartments hampers a full-scale assessment of the global surface and groundwater assets and introduces uncertainty in the understanding of how the water resources in the deep aquifer depend on the surface water availability, and *vice versa*. To improve assessments of the way surface water and groundwater are ultimately coupled and how they influence each other, datasets should provide both the soil moisture and depths of the water tables including those that form temporarily. Cross-validations against multiple independent datasets can provide reliable references to make interpretations, especially where observations are limited.

To overcome these limitations, we produced the SOIL-WATERGRIDS dataset, which was constructed by re-analysis of existing global scale datasets and by coupling these datasets with the BRTSim (BioReactive Transport Simulator^20^) mechanistic model for the geophysical flow of water. BRTSim was fed with globally gridded soil physical and hydraulic properties, land cover and use characteristics, and hydrometeorological data harmonised to a resolution of 0.25 × 0.25 degree per grid cell (about 30 km at the equator). BRTSim resolves the variably-saturated water flow over a vertical profile from land surface to 56 m depth and covers a time frame of 45 years from 1970 to 2014. We validated the SOIL-WATERGRIDS modelling estimates of volumetric soil water content against globally-gridded satellite data (ESA/CCI^21–23^), modeling reconstructions and re-analyses (GLDAS^19,24,25^), and distributed measurements (ISMN network^18,26^). We validated the water table depth estimates against selected groundwater data in^10^. These validations are also used to determine the data quality index of SOIL-WATERGRIDS. In addition to those validations, we benchmarked the location of ponding occurrence against the SWAMPS dataset for wetlands^27^ and our estimates of surface runoff against re-analyses in the GRUN dataset^28^.

## Methods

### Seeding databases and data pre-processing

For the development of SOIL-WATERGRIDS, we deployed the BRTSim model^20^ on a global grid constructed using existing georeferenced data. Details of all the datasets are summarised in Online-only Table 1 and described below.

The seeding data for the soil physical properties include the soil texture (sand, silt, and clay fractions) and bulk density from SoilGridsv2.0^29^, and porosity from SoilGridsv1.0^30^. The soil hydraulic properties include the permeability, the pore volume distribution index, and the air-entry suction of the Brooks and Corey model^31,32^, and the residual soil water content^33^. The land cover type in year 2014 was taken from MODIS/IGBP 2019^34,35^, while the ecosystem-specific root depth profiles for crops and native ecosystems are from^36,37^, respectively. The water security indicators and crop calendars^38–40^ were used to estimate the irrigation volumes as in^36^. The seeding hydrometeorological data include the historical global daily precipitation from the Climatic Research Unit gridded Time Series dataset from 1970 to 2018 (CRU/TS^41^), the daily actual evapotranspiration from the Global Land Evaporation Amsterdam Model from 1980 to 2017 (GLEAM^24,25^), the monthly potential evapotranspiration from the CRU/TS, and the monthly snowmelt data from Global Land Data Assimilation System (NOAH/GLDAS^19^). We also used the soil water content and saturation of the NOAH/GLDAS Version 2, satellite data from the European Space Agency, Climate Change Initiative for Soil Moisture (hereafter ESA/CCI^21–23^), data-assimilation soil moisture from the Global Land Evaporation Amsterdam Model (GLEAM, Martens *et al*., 2017^25^), the ground-based soil moisture measurements from the International Soil Moisture Network (ISMN^18,26^), and the long-term average and long-term monthly averages of the water table depth in^10^. Finally, we used the SWAMPS dataset for wetlands^27^ and the monthly total surface runoff from the GRUN dataset for additional benchmarking^28^.

The workflow used to generate the SOIL-WATERGRIDS is represented in the Supplementary Information, Figure S1. Heterogeneous seeding datasets were first harmonised to the chosen resolution of 0.25 × 0.25 degree per grid cell, which corresponds to about 30 × 30 km at the equator. The resolution harmonisation was conducted with a conservative (phycnophylactic) linear interpolation for the quantities explicitly representing mass or mass fluxes (e.g., precipitation, evapotranspiration, and others), or with a simple interpolation otherwise (e.g., soil physical properties, and others). Conservation of the quantity of interest was achieved by first calculating the areal integral at the original resolution and by rescaling the resized data to the areal integral. Data resizing was conducted for either type of variables by linear interpolation with the *imresize* function in @Matlab2019b. This method achieved conservation of mass and average values in all climatic regions with a percent relative error smaller than 0.01% (see sample validation in Supplementary Information, Figure S2). Harmonised data were used in intermediate steps for data reconstruction and calculation of the surface runoff and water budget in each grid cell.

Time series reconstruction was implemented for the GLEAM actual evapotranspiration (*ETA*) in years 1970 to 1979 because data in those years are not available. To this end, we used the potential evapotranspiration (*PET*) of the CRU/TS datasets. Because *ETA* and *PET* are from two independent sources, we tested data consistency by counting the number of times *ETA* is greater than 1.2 *PET*, where 1.2 is the excess evapotranspiration in cropping systems identified by the FAO method^38^. We found inconsistencies in 5.8 to 7.4% of grid cells within the computational domain across years from 1980 to 2014 (Supplementary Information, Figure S3), where both *ETA* and *PET* exist, but we have not applied adjustments to the original or harmonized data. The actual monthly evapotranspiration, *ETA*_*m,i*_ (mm month^−1^), in month *m* and grid cell *i* was reconstructed as

1 $$ET{A}_{m,i}={r}_{m,i}\times PE{T}_{m,i}$$

where *PET*_*m,i*_ is the monthly potential evapotranspiration in grid cell *i* available from CRU/TS and *r*_*m,i*_ is the ratio of the long-term monthly average actual evapotranspiration $\overline{ET{A}_{m,i}}$ to the long-term monthly average potential evapotranspiration $\overline{PE{T}_{m,i}}$ in the same month *m* and grid cell *i* throughout the years where both datasets exist. Comparison of *ETA* reconstructed using Eq. (1) and the long-term monthly means available from the GLEAM dataset returned R = 0.99 and *p* < 0.01 (Supplementary Information, Figure S4). The actual evapotranspiration used as a boundary condition in BRTSim can be interpreted as a potential evapotranspiration flux because water can only be removed by the plant root system if water is available at a saturation above residual.

The daily snowmelt rate *SM*_*i*_ (mm day^−1^) in each grid cell *i* was calculated from the available monthly *SM*_*m,i*_ estimates in NOAH/GLDAS and was next combined with the daily precipitation *P*_*i*_ to calculate the daily surface runoff *Q*_*i*_ (mm day^−1^) in each grid cell *i* following the Curve Number method (NRCS-CN^42^) as

2 $${Q}_{i}=\frac{{\left({P}_{i}+S{M}_{i}-{I}_{a,i}\right)}^{2}}{{P}_{i}+S{M}_{i}-{I}_{a,i}+{S}_{i}}$$

3 $${S}_{i}=\frac{25,400}{C{N}_{i}}-254$$

where ${I}_{a,i}=0.2{S}_{i}$ is the initial abstraction, *S*_*i*_ is the maximum water retention capacity of the soil before releasing water, and *CN*_*i*_ is the curve number at *fair* moisture conditions tabulated in the NRCS-CN technical report as a function of soil type, hydrological characteristics, and land use. In addition to these classifications, the normalized antecedent precipitation index (*NAPI*^43^,) was introduced to calculate the soil moisture based on the precipitation in the previous days as

4 $$NAPI=\frac{{\sum }_{t=-1}^{{T}_{p}}P(t){k}_{CN}^{-t}}{{P}_{avg}{\sum }_{t=-1}^{{T}_{p}}{k}_{CN}^{-t}}$$

where *T*_*p*_ = 5 is the number of antecedent days recommended in the NRCS-CN, *k*_*CN  *_=0.85 is the decay constant of the soil moisture over time^44^, *P(t)* is the precipitation at day *t, and* P_*avg*_ is the annual average precipitation. *NAPI* changes over time and in each grid cell. Soil in a grid cell is considered “dry” if *NAPI* < 0.33, “wet” if *NAPI* > 3, and at “fair condition” otherwise. Finally, the precipitation dependent curve number *CN*_*i*_ in Eq. (3) was written as

5 $$C{N}_{i}^{d}=\frac{C{N}_{i}}{2.281-0.0128C{N}_{i}};C{N}_{i}^{w}=\frac{C{N}_{i}}{0.427-0.00573C{N}_{i}}$$

where superscripts *d* and *w* indicate dry and wet conditions, respectively. The runoff flow direction was derived from the steepest down-slope gradient calculated from the digital elevation map^45^. Finally, the travel time of *Q*_*i*_ from the source grid cell *i* to the recipient grid cell was calculated as the time of concentration in days *T*_*c,i*_ using the watershed lag method of the NRCS-CN as

6 $${T}_{c,i}=\frac{1}{24}\frac{{L}_{i}^{0.8}{\left({S}_{i}+1\right)}^{0.7}}{1,140{Y}_{i}^{0.5}}$$

where *L*_*i*_ is the distance in feet between the centroids of the source grid cell and its recipient, and *Y*_*i*_ is the average slope between the source and recipient grid cells. In our implementation, *T*_*c,i*_ is specific to each grid cell and is used to allocate the fraction of generated runoff, ${f}_{Q}=min\left\{1,1/{T}_{c}\right\}$, eventually leaving the source grid cell in a day. The remaining generated runoff, (1-*f*_*Q*_)*Q*, is applied on the subsequent day using again *f*_*Q*_; this procedure is repeated until 99.9% of the initial *Q*_*i*_ is allocated through time. The daily runoff rate *Q*_*i*_ is not subtracted from *P*_*i*_ and *SM*_*i*_ but is applied as an outlet flux from the topsoil of grid cell *i*. We recall that the NRCS-CN method has limitations on the accounting of snowmelt, the aspect that we address in “Technical Validation”.

Finally, the calculation of the daily irrigation rate consisted in identifying the grid cells where irrigation is applied using the crop water security indicators in^39^, while the crop calendars in^40^ were re-interpreted to calculate the daily gridded normalised crop calendar and identify the crop irrigation period^36^. In an irrigated grid cell, the daily irrigation rate is calculated as the gap ${I}_{i}=f\left(1.5ET{A}_{i}-{P}_{i}\right)$ between precipitation *P*_*i*_ and actual evaptranspiration *ETA*_*i*_ during the crop irrigation period with *f* = 0.8 or *f* = 0.2 depending on whether irrigation is major or minor, respectively, according to the classification in^39^.

The hydrometeorological data were used to estimate the decadal water balance, *W*_*i*_, in grid cell *i* from 1970 to 2014 as

7 $${W}_{i}=\sum {P}_{i}-\sum ET{A}_{i}+\sum S{M}_{i}+\sum {Q}_{i}+\sum {I}_{i}$$

where the terms on the right-hand side are the cumulative precipitation, actual evapotranspiration, snowmelt, net surface runoff (input minus output), and irrigation in each decade and in each grid cell. The irrigation term, *I*, is only accounted for in grid cells where the water table depth is below the depth of our computational domain of 56 m. This is because we assumed irrigation abstractions to occur from within the computational domain in grid cells with a water table above 56 m depth, and therefore the water abstraction from the aquifer and applied at land surface was not accounted for in the water budget of Eq. (7) as water is recirculated. Equation (7) is used to condition the boundary flows in each grid cell with an additional flow of water lasting for each decade of the reference period (positive or negative depending on the grid cell) to prevent the soil column defined by finite volumes in BRTSim to become fully saturated or fully depleted. Inclusion of this decade-specific water balance flow does not affect the intra day and intra seasonal fluctuations in soil water content and water table depth, and compensates for the horizontal groundwater flow not explicitly included in our modeling (see also Section “BRTSim modelling and outputs”). Estimates in SOIL-WATERGRIDS do not explicitly account for river flow discharge within a watershed and only include an approximation to the snowmelt flow into runoff. An analysis and benchmarking of our approach and estimates against existing runoff data is expanded in Section “Technical Validation”.

### Grid cell selection, design, and initialisation

The bounding box of SOIL-WATERGRIDS extends between longitudes −180°W and 180°E and between latitudes −55.5°S and 70.5°N. A total of about 168,000 grid cells of interest were selected according to the following criteria: (1) we excluded grid cells with missing data using the information provided in the seeding datasets (see Section “Seeding databases and data pre-processing”); (2) we excluded grid cells above the permanent snow line at 3,500 m elevation using the digital elevation map in^45^ and grid cells with more than 10% permafrost area using the NEO/NASA data in^46^ to reduce biases caused by frozen soil, which is not explicitly described in our modelling and for which no data exist for validating the water table depth and surface soil moisture; and (3) we excluded grid cells with more than 10% wetland area based on the MODIS/IGBP land cover classification because we did not explicitly model wetlands hydraulics in this work even if we track the occurrence of ponding in our computational grid (more detail on ponding is provided in Section “Technical Validation”). Runoff water from and to grid cells neighbouring the computational domain was explicitly accounted for to minimise biases on water budget at the boundary of the computational domain. Grid cells included in the computational domain are represented in Figure S5 of Supplementary Information.

**Fig. 1:**
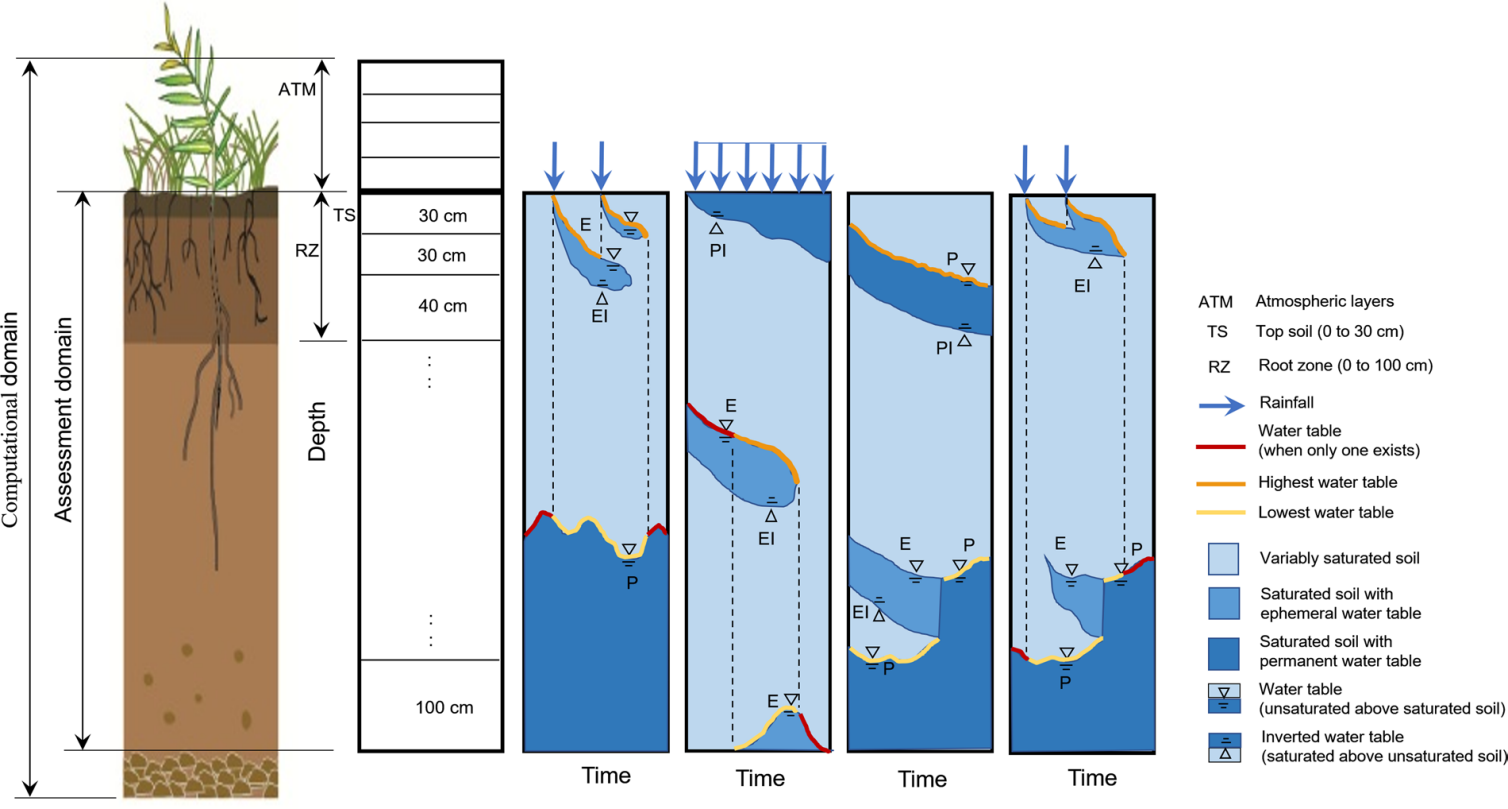
Computational and assessment domains (**a**), and their discretisation in the BRTSim modelling (**b**) with examples of water table types (**c** to **f**). Letters P and E indicate “permanent” and “ephemeral” water tables, the former existing for the entire assessment period and the latter existing for a shorter period. The additional letter I associated to E and P (i.e., PI and EI) indicates inverted water tables. Water tables marked in yellow and orange are the lowest and highest, respectively, while those in red indicate cases when only one water table exists. The water tables marked with these colors are distributed in SOIL-WATERGRIDS. The assessment period is from 1970 to 2014. Drawing of the soil profile on the left is elaborated from https://www.qld.gov.au/environment/land/management/soil/soil-explained/forms.

The computational grid was designed to describe an archetype 56 m deep soil column with a sequence of 64 finite volumes with varying thickness vertically stratified in each grid cell (Fig. 1a,b). The top soil (TS, 0 to 30 cm) consists of the first soil layer of 30 cm thickness; the root zone (RZ, 0 to 100 cm) is defined by the first three soil layers of 30, 30, and 40 cm thickness; the layers from 1 to 3 m depth have 40 cm thickness, from 3 to 6 m depth have 50 cm thickness, and from 6 to 56 m depth have 1 m thickness. We included four atmospheric layers of 30 cm thickness above the soil to allow for occasional water ponding.

The soil textural properties (sand, silt, and clay fractions), bulk density, and porosity for each soil layer from the surface to 2 m depth were assigned using data in SoilGrids^30^ interpolated at the depths describing the soil column in our computational domain. The soil properties below 2 m depth were assigned using the values of the closest upper known depth. The soil permeability, *k*, pore volume distribution index, *b*, and air-entry suction, *ψ*_*s*_, of the Brooks and Corey model were assigned using data in^32^ for all soil layers from the surface down to 2 m depth after interpolation at the depths of our computational domain. Below 2 m depth, the permeability, *k*, was calculated with a negative exponential function of the depth, *z*, similar to the approach in^47^

8 $$k={k}_{2m}\;{e}^{-z/\beta }$$

where *k*_2*m*_ is the permeability at 2 m depth and *β* is an empirical parameter calculated assuming that the permeability *k*_55.5*m*_ at 55.5 m depth (i.e., the center of mass of the deepest layer in our discretisation) is one-tenth of *k*_2*m*_ after trial and error testing on randomy selected grid cells, hence, $\beta ={{\rm{z}}}_{55.5{\rm{m}}}/{\rm{ln}}(10)$ = 24.1 m. The hydraulic parameters *b* and *ψ*_*s*_ were calculated in each grid cell *i* assuming hydraulic equilibrium (i.e., the total hydraulic head is $H=\psi \left({S}_{\ell }\right)+z$ = const) at the depth *z*_*WTD*_ of the known water table and at the soil surface for the known soil saturation ${S}_{\ell }$. The known *z*_*WTD,i*_ in grid cell *i* was taken from^10^ while ${S}_{\ell }$ at the soil surface was initialised to the average value of data in GLEAM, NOAH/GLDAS, and ESA/CCI. The values of *b* and ${\psi }_{s}$ calculated in this way were next averaged with the globally-gridded values in^32^ at the depths between the soil surface and 2 m depth. Hence, the initial soil water saturation profile produced values at the surface close to available soil moisture datasets, and water table depths close to those in^10^. The liquid residual saturation, ${S}_{\ell r}$, from^47^ was therefore adjusted in some grid cells to satify the condition ${S}_{\ell r} < {S}_{\ell }$, while the gas residual saturation ${S}_{gr}=0.1$ was used in all soil layers and grid cells. An analysis of the actual values of *b* and ${\psi }_{s}$ used in our modeling show that corrections introduced here do not substantially skew their frequency distributions (Supplementary Information, Figure S6).

### BRTSim modelling and outputs

BRTSim-v4.1a is a general-purpose solver of reaction-advection-dispersion processes in variably-saturated soil systems. BRTSim uses a hybrid explicit-implicit finite volume scheme to solve the Richards equation^48^ and describes the variably-saturated water flow along the vertical soil profile with heterogeneous soil physical and hydraulic properties (details available in the User Manual and Technical Guide^20^). For SOIL-WATERGRIDS, the relative permeability-water potential-water saturation relationships of the Brooks-Corey model were used with parameters assigned as described in Section “Grid cell selection, design, and initialisation”. The core solvers in BRTSim are fundamentally one dimensional; however, the parallelisation implemented in this work allowed us to use it over the global grid as a pseudo three-dimensional solver accounting for a one-way horizontal water flow representing surface runoff.

The BRTSim modelling outputs were post-processed to calculate the monthly mean soil water saturation, ${S}_{\ell }$, and volumetric water content, *θ*, in the three layers of the root zone, and the water table depth, WTD, ranging from land surface to a depth of 50.5 m (corresponding to the center of mass of that soil layer), that is, the bottom five layers down to 56 m depth were excluded from assessment to reduce biases from boundary effects (Fig. 1b).

Relative to the water table, the solution of the variably-saturated Richards equation allowed us to describe dynamic changes along the vertical profile, including the formation of multiple water tables stratified at different depths. Our modelling outputs showed that up to 6 water tables occurred in a minor fraction of grid cells for short periods of time. After *a posteriori* inspection, we found that all water tables not known from prior information at the initialisation of our modelling assessment appeared after intense precipitation events and were generally located above the known water table. Hence, our assessment can include permanent water tables with variable depth but lasting for the entire duration of our assessment (marked with P in Fig. 1) and ephemeral water tables with variable depth (marked with E in Fig. 1). Inverted water tables, characterised by the saturated soil above the unsaturated soil, are associated with all instances in which ephemeral water tables develop (marked with EI, Fig. 1). Permanent inverted water tables (marked with PI in Fig. 1d) are highly unlikely over the 45 years of assessment within the geographic region of interest and we have not found occurrence of this type of water table. Ephemeral water tables can also raise if the bottom unsaturated soil layer becomes saturated for a short period of time. Both ephemeral and permanent water tables can co-exist at any time of our assessment and can also combine with each other or separate into multiple saturated lenses (Fig. 1e,f). Because our modelling can describe a wide variety of forming, persisting, and fading water tables, we identified and counted all water tables (P and E types) but, for simplicity, we distribute in SOIL-WATERGRIDS only the highest (closest to the surface) and lowest (deepest from the surface) when multiple water tables co-exist. SOIL-WATERGRIDS also identifies grid cells where ponding occurs. To the best of our knowledge, SOIL-WATERGRIDS is the only data product that releases these assessments of the water table dynamics.

## Data Records

The SOIL-WATERGRIDS distribution dataset includes NetCDF formatted files available for download from the *Zenodo* repository^49^. These files contain the variables described in detail in Table 1 with legends expanded in Table 2. In addition to the distribution dataset, we make available the full global-scale modelling inputs (deployable on a UNIX computer cluster) from the *Zenodo* repository^49^ as described in Section “Code Availability”. Additional information on both distributed dataset and modelling is available in the Technical Documentation in the *Zenodo* repository^49^.

**Table 1:** SOIL-WATERGRIDS data distribution files and variables. All files included in the data distribution are compressed and contained in file SOIL-WATERGRIDS_NC_Distributed.zip available from^81^  and^49^.

	File Name	Description	Variable		
			Name	Description	Unit
SOIL-WATERGRIDS_NC_Distributed.zip	SOIL-WATERGRIDS_**YYYY**.nc	Contains the globally gridded data in year **YYYY** (from 1970 to 2014) of the monthly mean soil water saturation in three layers of the root zone and the monthly mean depth of the highest and lowest water tables. ^(a)^When only one water table exists, these report the same value of water table depth.	Sl_0_30	Soil water saturation, 0 to 30 cm	[-]
			Sl_30_60	Soil water saturation, 30 to 60 cm	[-]
			Sl_60_100	Soil water saturation, 60 to 100 cm	[-]
			^(a)^WTDH	Depth of highest water table	[m]
			^(a)^WTDL	Depth of lowest water table	[m]
			WTDN	Number of water tables	[-]
			time	Month of the year	[-]
	SOIL-WATERGRIDS_ltm.nc	Contains the globally gridded long-term monthly mean soil water saturation in three layers of the root zone and the long-term monthly mean depth of the highest and lowest water tables	Sl_0_30_ltm	Soil saturation, 0 to 30 cm	[-]
			Sl_30_60_ltm	Soil saturation, 30 to 60 cm	[-]
			Sl_60_100_ltm	Soil saturation, 60 to 100 cm	[-]
			WTDH_ltm	Depth of highest water table	[m]
			WTDL_ltm	Depth of lowest water table	[m]
	SOIL-WATERGRIDS_qi.nc	Contains the globally gridded data quality index *QI* calculated as described in Section “Technical Validation”	QI	Quality index, 0 (worse) to 1 (best)	[-]
	SOIL-WATERGRIDS_ext.nc	Contains the soil porosity ($\phi$), air-entry suction (${\psi }_{s}$), pore volume distribution index (b), and and water residual saturation (*Slr*) in the three layers of the root zone. Data can be used use in combination with SOIL-WATERGRIDS_yyyy.nc and SOIL-WATERGRIDS_ltm.nc to calculate the volumetric soil water content and the soil water potential $\psi$ using the Brooks and Corey model.	phi_0_30 phi_30_60 phi_60_100	0 to 30 cm 30 to 60 cm 60 to 100 cm	[-] [-] [-]
			psis_0_30 psis _30_60 psis_60_100	0 to 30 cm 30 to 60 cm 60 to 100 cm	[m] [m] [m]
			b_0_30 b_30_60 b_60_100	0 to 30 cm 30 to 60 cm 60 to 100 cm	[-] [-] [-]
			Slr_0_30 Slr_30_60 Slr_60_100	0 to 30 cm 30 to 60 cm 60 to 100 cm	[-] [-] [-]
	Read_NC.m	Editable script written in Matlab 2019b to read and represent.NC files (example).

**Table 2:** Legend of variables in SOIL-WATERGRIDS data distribution.

Variable	Legend	
Sl_0_30, Sl_30_60, Sl_60_100	−2	Ocean
Sl_0_30_ltm, Sl_30_60_ltm, Sl_60_100_ltm	−1	No data
Slr_0_30, Slr_30_60, Slr_60_100	[0, 1]	Range of values
WTDL, WTDH, WTDL_ltm, WTDH_ltm	−2 −1 0 [0.15, 50.5] 100	Ocean No data Ponding Range of values Below 50.5 m depth
WTDN	−2	Ocean
	−1	No data
	[0, 6]	Range of values
QI	−2	Ocean
	−1	No data
	[0, 1]	Range of values, 1 is best
b_0_30, b_30_60, b_60_100	−2	Ocean
	−1	No data
	[1.2, 112]	Range of values
phi_0_30, phi_30_60, phi_60_100	−2	Ocean
	−1	No data
	[0.1, 0.80]	Range of values
psis_0_30, psis _30_60, psis_60_100	2	Ocean
	1	No data
	[−1.60,0]	Range of values

## Technical Validation

The quality of the distributed dataset is measured at two levels: the first, called validation, refers to the direct comparison of the target variables distributed in SOIL-WATERGRIDS against other existing data. The second, called benchmarking, refers to the comparison against other existing data of intermediate variables not distributed in the SOIL-WATERGRIDS dataset but instrumental to the estimates in SOIL-WATERGRIDS.

### Methods of validation and benchmarking

The volumetric soil water content *θ* in TS (0 to 30 cm) and RZ (0 to 100 cm), and water table depth WTD estimated in SOIL-WATERGRIDS were validated against existing independent data listed in Table 3 for the same variables. For both SOIL-WATERGRIDS and the validation datasets, we retrieved or calculated the long-term mean (LTM) and long-term monthly mean (mLTM) depending on data frequency. Validation against some datasets was limited to the available geographic regions such as for ESA/CCI, ISMN, and data in^10^. The metrics to measure the quality of SOIL-WATERGRIDS estimates consisted in the anomaly ${\Delta X}_{Y}=X-Y$ of the SOIL-WATERGRIDS variables *X* against the corresponding validation set *Y*, the normalised root mean square deviation NRMSD, the spatial and temporal Pearson’s correlations R, and the Duvellier coefficient λ accounting for data biases^50^. These metrics are defined in Table 4 and were calculated for either LTM, or mLTM, or both data depending on data availability. Of these metrics, ${\Delta X}_{Y}$ and R were used to calculate the data quality index *QI* as described later in Section “Data quality”.

**Table 3:** Target variables in SOIL-WATERGRIDS data distribution used for validation against existing datasets.

Variable	SOIL-WATERGRIDS	GLEAM	NOAH/GLDAS	^(a)^ESA/CCI	^(a)^ISMN	^(a)^Fan *et al*., (2017)
*θ* in TS	LTM, mLTM	LTM, mLTM	LTM, mLTM	LTM, mLTM	mLTM	
^(b)^*θ* in RZ	LTM, mLTM	LTM, mLTM	LTM, mLTM			
WTD	LTM, mLTM					LTM, mLTM

LTM and mLTM stand for long-term mean and long-term monthly mean either accessible in or calculated from the original datasets. ^(a)^ Grid cells with missing values in the source data were not paired to SOIL-WATERGRIDS. ^(b)^ The values of *θ* in RZ in SOIL-WATERGRIDS were calculated as averages of *θ* at the three soil depths defined in our computational domain weighted by the corresponding soil layer thicknesses.

**Table 4:** Definition of metrics measuring the quality of SOIL-WATERGRIDS data distribution.

Quantity	Metrics used with the long-term mean (LTM)	Metrics used with the long-term monthly means (mLTM)
**Anomaly**	^(a)^ $\Delta {X}_{Y}\left(i\right)=X\left(i\right)-Y\left(i\right)$	$\Delta {X}_{Y}(i,m)=X(i,m)-Y(i,m)$
**Normalised root mean square deviation**		${\rm{NRMSD}}(m)=\frac{\sqrt{\frac{1}{N}{\sum }_{i}^{N}\Delta {X}_{Y}{(i,m)}^{2}}}{[max{\{Y(m)\}}_{s}-min{\{Y(m)\}}_{s}]}$
**Spatial Pearson’s correlation coefficient**	${R}_{S}$	${R}_{S}(m)$
**Temporal Pearson’s correlation coefficient**		^(a)^ ${R}_{T}\left(i\right)$
**Duvellier coefficient**		$\lambda \left(m\right)=\alpha {R}_{S}\left(m\right)$ with $\alpha =2{\left[\frac{{\sigma }_{X}{\left(m\right)}_{s}}{{\sigma }_{Y}{\left(m\right)}_{s}}+\frac{{\sigma }_{Y}{\left(m\right)}_{s}}{{\sigma }_{X}{\left(m\right)}_{s}}+\frac{{\left(\overline{X{\left(m\right)}_{s}}-\overline{Y{\left(m\right)}_{s}}\right)}^{2}}{{\sigma }_{X}{\left(m\right)}_{s}{\sigma }_{Y}{\left(m\right)}_{s}}\right]}^{-1}$

Estimates *X* of SOIL-WATERGRIDS are compared to the corresponding independent datasets *Y* referred to as in Table 1. The indices *i* and *m* indicate the grid cells and the month, respectively; the subscripts *S* and *T* indicate spatial and temporal operators, respectively; $\sigma$ indicates the variance; the bar above symbols indicates the average. ^(a)^ validation metrics used for calculation of the data quality index *QI* described in Section “Data quality”.

We benchmarked intermediate data not directly distributed with SOIL-WATERGRIDS. Specifically,the geographic occurrence of ponding was benchmarked against the SWAMPS wetlands dataset, while the calculated runoff and water balance were benchmarked against the GRUNv1 dataset. These benchmarking were not included in the data quality index as ponding and runoff are not the target variables distributed in SOIL-WATERGRIDS, but further supported our working approach in a quantitative way.

### Validation of the soil water content

We compared the long-term mean volumetric water content *θ* in TS against available re-analyses from NOAH/GLDAS and GLEAM, satellites data provided by ESA/CCI, and ground-logged readings from 39 networks and a total of 1,766 data points from 28 countries of the ISMN covering a maximun time span from 1952 to 2014 (Table 5). A similar comparison was repeated for *θ* in RZ against the corresponding quantity in the GLEAM and NOAH/GLDAS datasets.

**Table 5:** ISMN networks and corresponding total and selected (in parenthesis) number of stations used in this work.

Network	Number of stations	Reference	Network	Number of stations	Reference
AACES	49 (49)	^54^	ORACLE	6 (6)	N/A
AMMA-CATCH	7 (7)	^55^	OZNET	38 (38)	^56^
ARM	35 (22)	N/A	PBO_H2O	159 (146)	^57^
AWDN	50 (50)	N/A	PTSMN	20 (20)	^58^
BNZ-LTER	12 (12)	^59^	REMEDHUS	24 (23)	N/A
CARBOAFRICA	1 (1)	^60^	RISMA	24 (23)	^81^
CHINA	57 (40)	^61^	RSMN	20 (19)	N/A
COSMOS	109 (11)	^62^	RUSWET-AGRO	212 (53)	^63^
CTP_SMTMN	57 (57)	^64^	RUSWET-GRASS	122 (76)	^63^
DAHRA	1 (1)	^65^	SASMAS	14 (14)	^66^
FLUXNET	2 (2)	N/A	SCAN	239 (229)	N/A
FMI	27 (27)	N/A	SMOSMANIA	23 (22)	^67^
FR_Aqui	5 (5)	N/A	SNOTEL	441 (437)	^68^
HOBE	32 (2)	^69^	SOILSCAPE	171 (165)	^70^
ICN	19 (18)	^71^	TERENO	5 (5)	^72^
IPE	2 (1)	N/A	UMBRIA	13 (13)	^73^
iRON	9 (9)	^74^	USCRN	115 (115)	^75^
LAB-net	3 (1)	^76^	USDA-ARS	4 (4)	^77^
MONGOLIA	44 (43)	^63^			

Of the original 65 networks and approximately 2678 stations, selection was based on (i) at least 4 consecutive years were available for a station and (ii) data quality was reported in the original dataset as “good”. Stations located in the same grid cell of our computational domain were averaged.

**Fig. 2:**
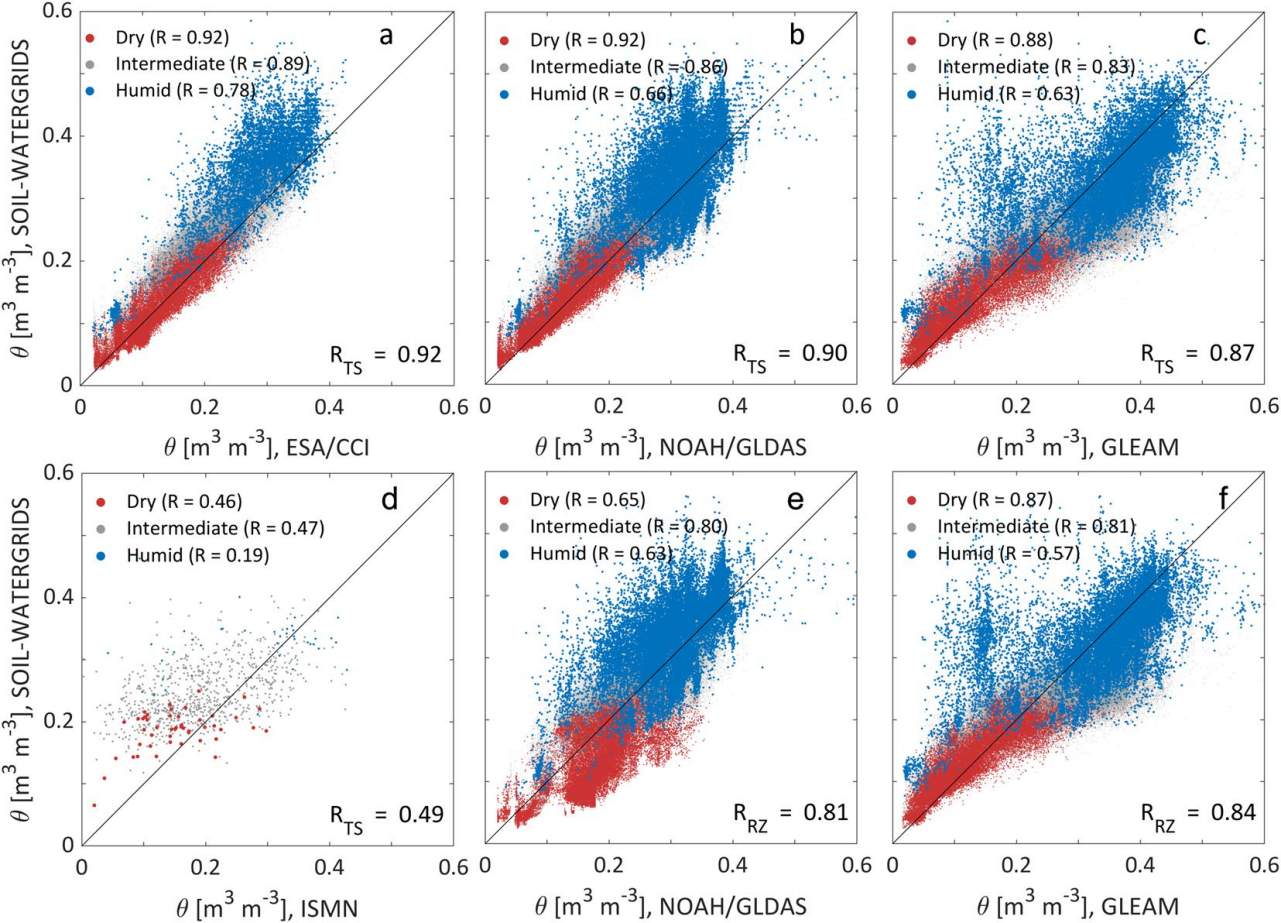
(**a**) to (**d**) Long-term mean estimates in volumetric water content *θ* in the top soil (TS, 0 to 30 cm) and (**e**) to (**f**) the root zone (RZ, 0 to 100 cm) against datasets from ESA/CCI, NOAH/GLDAS, GLEAM, and ISMN, respectively. The long-term mean values of SOIL-WATERGRIDS are calculated over the assessment period 1970–2014. Dry and humid regions were identified by grid cells where *θ* in the top soil is below 0.8 × ${\theta }_{FC}$ and above ${\theta }_{FC}$, respectively, for 75% of the time within the 45 years of assessment, with ${\theta }_{FC}$ the water content at field capacity corresponding to a suction *ψ* = −33 kPa. A map of the geographic distribution of dry and humid regions is available in Supplementary Information, Figure S7.

The spatial Pearson’s correlation between SOIL-WATERGRIDS estimates and the validation datasets is R > 0.87 in TS (Fig. 2a–c) except in relation to ISMN, which scores R = 0.49 (Fig. 2d). In RZ, R > 0.81 (Fig. 2e,f). Figure 2 also highlights how SOIL-WATERGRIDS aligns with the validation datasets in dry (red markers), intermediate (gray markers) and humid (blue markers) regions, where dry and humid regions are identified by *θ* < 0.8×${\theta }_{FC}$ and *θ* > ${\theta }_{FC}$, respectively, for 75% of the time within the 45 years of assessment, with ${\theta }_{FC}$ the water content at field capacity corresponding to *ψ* = −33 kPa suction (map of identified dry and humid regions is shown in Supplementary Information, Figure S7). Nonetheless, we found a few grid cells identified as humid regions that showed relatevely low *θ* values in TS, which can result from a combination of hydraulic parameters *b* and ${\psi }_{s}$ falling in the tails of their probability distribution in those climatic areas (see also Supplementary Information, Figure S6). We note also that the spatial correlation in humid regions was generally lower in both TS and RZ than in intermediate and dry regions. This may be caused by soil water dynamics difficult to fully capture in the equatorial regions in the Amazon and South east Asia, where most wet regions were identified, or in boreal forests that experience soil frosting even if only grid cells where permafrost is less than 10% of their area were included in our assessment.

The long-term mean *θ* in TS estimated in SOIL-WATERGRIDS is well in agreement with the ESA/CCI, NOAH/GLDAS and GLEAM, with 95% of the grid cells having an anomaly ranging between −0.06 and 0.08 m^3^ m^−3^ (Supplementary Information, Figure S8). SOIL-WATERGRIDS generally overestimates the ISMN observations with 95% of the grids cells having anomalies between −0.06 and 0.17 m^3^ m^−3^. In RZ, 53% of grids cells in SOIL-WATERGRIDS underestimates the NOAH/GLDAS and GLEAM datasets by an average of −0.11 m^3^ m^−3^ while the remaining cells overestimated them only slightly (0.004 m^3^ m^−3^). These anomalies are distributed heterogeneously, but with geographic patterns such as lower *θ* in the northern hemisphere and slightly higher *θ* in some arid regions as compared to the validation datasets (Supplementary Information, Figure S8). Those anomaly patterns and their variability across datasets obtained by data reconstruction, modeling, reanalysis or their combination, suggest that SOIL-WATERGRIDS and those datasets may suffer from some biases relative to each other, but with the additional note that, among all datatsets, SOIL-WATERGRIDS is the only one to be constrained to both the volumetric water content in TS and RZ, and the water table depth (see Section “Validation of the water table depth”)

**Fig. 3:**
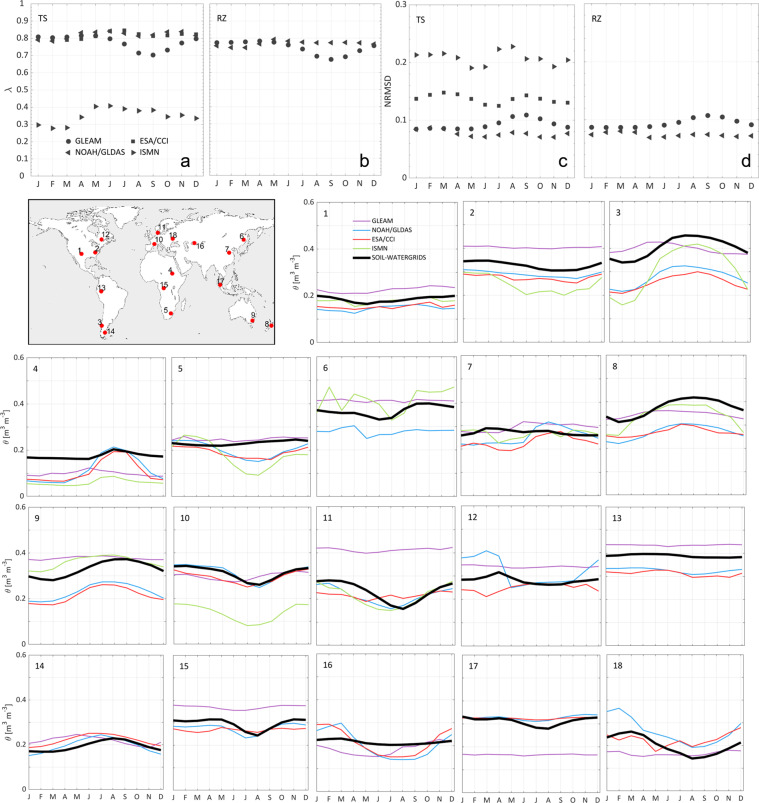
Seasonality assessment. (**a**) to (**d**) Duvellier coefficient λ and normalized root mean square deviation (NRMSD) calculated for the long-term monthly mean volumetric water content *θ* in the top soil (TS, 0 to 30 cm) and root zone (RZ, 0 to 100 cm) of SOIL-WATERGRIDS relative to the GLEAM, ESA/CCI, NOAH/GLDAS, and ISMN validation data. λ and NRMSD are calculated as described in Section “Technical Validation”, Tables 3 and 4. (map) geographic location of 18 randomly selected grid cells and (numbered panels) corresponding long-term monthly mean volumetric water content *θ* in the top soil (TS, 0 to 30 cm) estimated in SOIL-WATERGRIDS as compared to the validation datasets throughout the entire assessment period from 1970 to 2014.

The seasonality in the long-term monthly mean *θ* in TS of SOIL-WATERGRIDS captured relatively well the seasonality in ESA/CCI, NOAH/GLDAS, GLEAM, and ISMN (see 18 randomly sampled grid cells in Fig. 3). Globally, the long-term monthly means in TS and RZ do not present excessive biases, with λ > 0.7 (best value is λ = 1) and NRMSD < 0.15 except in relation to ISMN data that score lower λ and R values (Fig. 3a–d). However, some caveats regarding the comparison with the ISMN dataset influenced our validation: in fact, the SOIL-WATERGRIDS resolution does not allow for a direct comparison of single stations and, therefore, the average of the *in-situ* measurements within the corresponding grid cell in SOIL-WATERGRIDS may not represent the grid cell-scale average. Also, the data recorded in the ISMN have different temporal coverage, thus suggesting that the long-term means in SOIL-WATERGRIDS may not fully reproduce the actual temporal variation in those specific regions.

The seasonality in the monthly estimates of *θ* in TS of SOIL-WATERGRIDS throughout the entire period of assessment from 1970 to 2014 is generally highly and positively correlated with *θ* values averaged across the ESA/CCI, NOAH/GLDAS, and GLEAM datasets, with only minor regions of weak anti-correlation in very humid areas in the Amazon and very arid areas in the Sahara and Gobi deserts (Supplementary Information, Figure S9).

Finally, maps of the long-term average volumetric soil water content in TS and RZ throughout the assessment period from 1970 to 2014 are represented in Supplemenatry Information, Figure S10.

### Validation of the water table depth

**Fig. 4:**
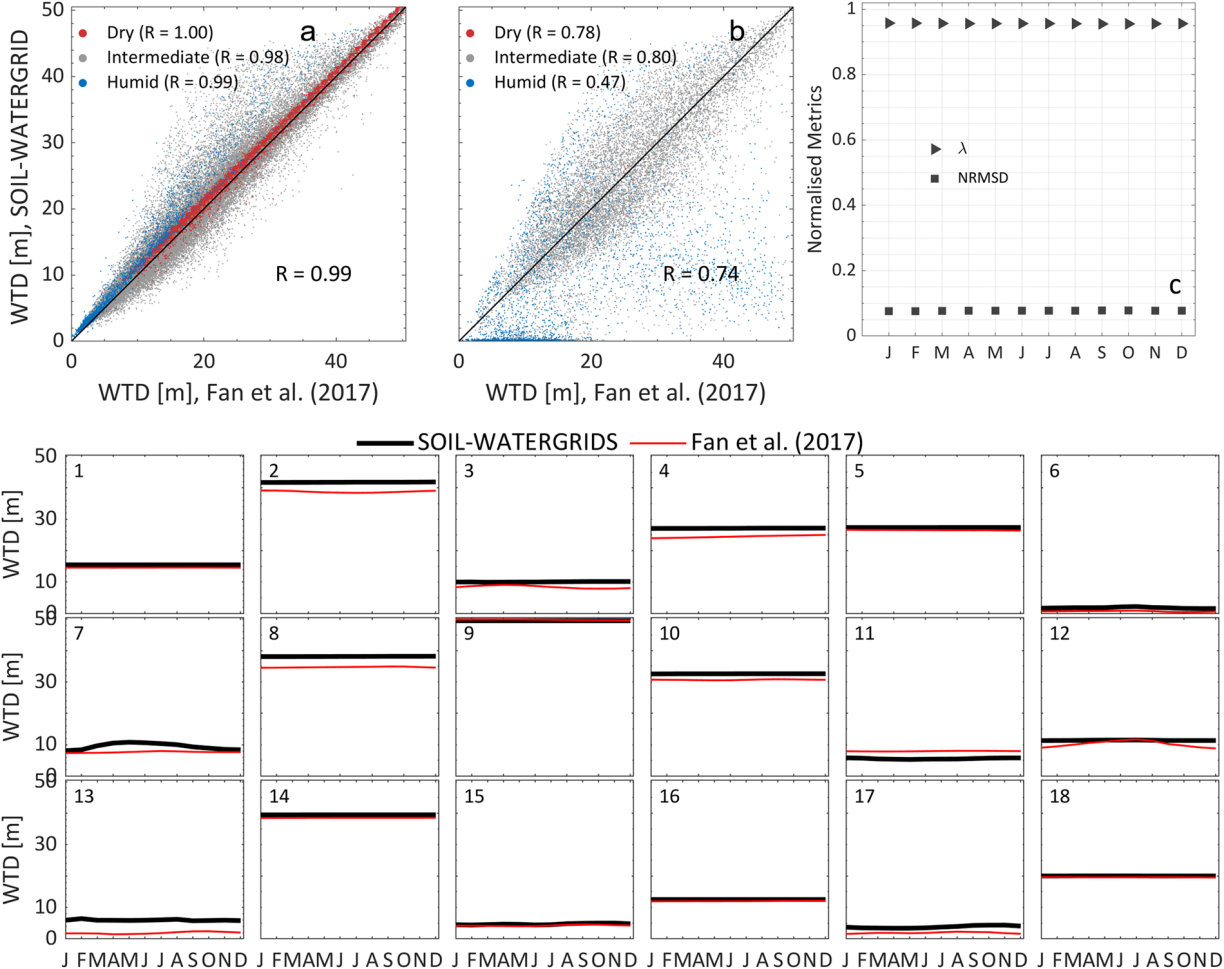
(**a**) and (**b**) scatterplot of SOIL-WATERGRIDS estimated WTD against data in^10^ when single and multiple water tables exist, respectively. When more than one water table existed in SOIL-WATERGRIDS, we used the water table closest to those in^10^. (**c**) Duvellier coefficient λ and normalized root mean square deviation (NRMSD) calculated for the long-term monthly mean WTD of SOIL-WATERGRIDS estimates against data in^10^. λ and NRMSD are calculated as described in Section “Technical Validation” and Table 4. Dry and humid regions were identified by grid cells where *θ* in the top soil is below 0.8 × ${\theta }_{FC}$ and above ${\theta }_{FC}$, respectively, for 75% of the time within the 45 years of assessment, with ${\theta }_{FC}$ the water content at field capacity corresponding to a suction *ψ* = -33 kPa. A map of the geographic distribution of dry and humid regions is available in Supplementary Information, Figure S7.

The long-term mean and long-term monthly mean of the water table depth (WTD) estimated in SOIL-WATERGRIDS were compared with data in^10^ using the same metrics described earlier. We excluded from validation about 1% of grid cells where values in^10^ are outside of our computational depth and where WTD in SOIL-WATERGRIDS are beyond the bottom boundary zone (below 50.5 m depth) for more than 10% of the assessment period. For validation of grid cells where more than one water table existed in SOIL-WATERGRIDS, we used the water tables closest to those in^10^. The SOIL-WATERGRIDS estimates of the long-term mean depth of water table matched those in^10^ reasonably well, with a high spatial correlation between the two datasets when both single and multiple water tables were present (R = 0.99 and R = 0.74, respectively, Fig. 4a,b). However, data dispersion was generally higher in grid cells with multiple water tables than all other grid cells (Fig. 4b), likely because of interactions between water tables such as anticipated in Section “BRTSim modelling and outputs”. We did not find substantial biases relative to dry and humid regions (represented in Supplementary Information, Figure S11a) with the exception of some regions where multiple water tables exist, which SOIL-WATERGRIDS tends to estimate closer to surface (Fig. 4b). The goodness of fit of the long-term monthly mean was characterized by λ > 0.92 and NRMSD < 0.08 (Fig. 4c). The average anomaly ranged between −5.0 and 4.4 m, which is slightly above our grid resolution in the vertical direction (Supplementary Information, Figure S11b).

The maps of the maximum number of water tables and the long-term average water table depth closest to data in^10^ throughout the assessment period from 1970 to 2014 are represented in Supplementary Information, Figure S12.

### Benchmarking the geographic distribution of ponding

The geographic location of the long-term mean WTD estimated in SOIL-WATERGRIDS was compared against the location of the long-term mean wetland area fraction in the SWAMPS dataset to determine if the grid cells where we identified ponding coincide with those of wetlands. We considered a grid cell to contain wetlands only if the maximum wetland area fraction reported in SWAMP from 2000 to 2014 exceeded a monthly mean of 5%. About 11% of the grid cells in SOIL-WATERGRIDS show occasional ponding and 73% of those overlap with those in SWAMPS (Supplementary Information, Figure S13). Ponding in SOIL-WATERGRIDS underestimated 36% of the wetlands in SWAMPS particularly across the Australian drylands, sub Saharan region, central east Europe, and Northwest Canada. However, arid regions in the Australian and sub Saharan drylands can flood during the rainfall season only occasionally when the soil dryness does not allow fast infiltration of rainwater, and the corresponding grid cells in SOIL-WATERGRIDS may have been excluded from comparison with SWAMPS. In fact, the frequency of occurrence of wetlands in those areas is 2 or 3 times per year and covers 20% of the total area at the most. Similarly, northern latitudes are characterized by widespread peatland areas with the water table mostly below the soil surface^51^ even if they are accounted for as wetlands in SWAMPS, and this may explain why SOIL-WATERGRIDS does not detect ponding in those regions.

### Benchmarking the runoff flow rate

We compared our estimates of surface runoff *Q*_*i*_ calculated using the NRCS-CN method against the monthly total runoff from the surface, groundwater, and river discharge from 1970 to 2014 available in the GRUNv1 dataset. Because GRUNv1 accounts for fluxes excluded in SOIL-WATERGRIDS (i.e., river flow discharge and groundwater flow), we first verified whether GRUNv1 data are the upper bound for the surface runoff in SOIL-WATERGRIDS (i.e., ${Q}_{i} < {Q}_{i,{\rm{GRUN}}}$) and we found that runoff *Q*_*i*_ estimated in this work is less than in GRUN in about 83% of grid cells (R = 0.30, Supplementary Information, Figure S14a). We next verified whether our hypothesis that the water balance *W*_*i*_ in Eq. (7) eventually compensated for unaccounted water fluxes at the surface or within the aquifer (i.e., ${Q}_{i}+{W}_{i}\approx {Q}_{i,{\rm{GRUN}}}$) and we found SOIL-WATERGRIDS estimates are nearly equally distributed around GRUNv1 data (R = 0.65, Supplementary Information, Figure S14b). We concluded that known limitations in the application of the NRCS-CN method in relation to snowmelt and the time of concentration, and the unaccounted river flow discharge and groundwater flow were fairly moderated by the decadal water balance *W*_*i*_ in our approach.

### Data quality

To calculate the data quality index *QI* of SOIL-WATERGRIDS, we considered two quality factors, *QF*_*Δ*_ and *QF*_*R*_, which account for the anomaly and temporal Pearson’s correlation against validation data, respectively.

*QF*_*Δ*_ describes the quality relative to the anomalies of SOIL-WATERGRIDS estimates in the validation of long-term mean soil water content *θ* in TS and RZ, and the long-term mean depth of water table. *QF*_*Δ*_(*i*) is calculated in each grid cell *i* as

9 $$Q{F}_{\Delta }\left(i\right)=\frac{1}{{n}_{i}}\sum _{{n}_{i}}[1-\delta {X}_{Y}^{{n}_{i}}\left(i\right)]$$

where $\delta {X}_{Y}^{{n}_{i}}(i)=\left|\Delta {X}_{Y}^{{n}_{i}}\left(i\right)\right|/max\{\Delta {X}_{Y}^{{n}_{i}}(i)\}$is the normalised anomaly for variable ${X}^{{n}_{i}}$ with $\Delta {X}_{Y}^{{n}_{i}}(i)$ the anomaly defined as in Table 4 in grid cell *i* against the dataset ${Y}^{{n}_{i}}$, with the number ${n}_{i}$ of assessable variables in grid cell *i* ranging between 4 and 7 depending on data availability (Table 3).

*QF*_*R*_ describes the quality relative to the temporal Pearson’s correlation of the long-term monthly mean soil water content *θ* in TS and RZ, and WTD of SOIL-WATERGRIDS estimates against the corresponding validation datasets. *QF*_*R*_(*i*) in grid cell *i* is calculated as

10 $$Q{F}_{{\rm{R}}}\left(i\right)=\frac{1}{{n}_{i}}{\sum }_{{n}_{i}}\frac{{R}_{T}^{{n}_{i}}\left(i\right)+1}{2}$$

where the argument in the sum on the right-hand side is the normalised Pearson’s coefficient ${R}_{T}^{{n}_{i}}\left(i\right)$ defined in Table 4 and ranging between 0 and 1.

**Fig. 5:**
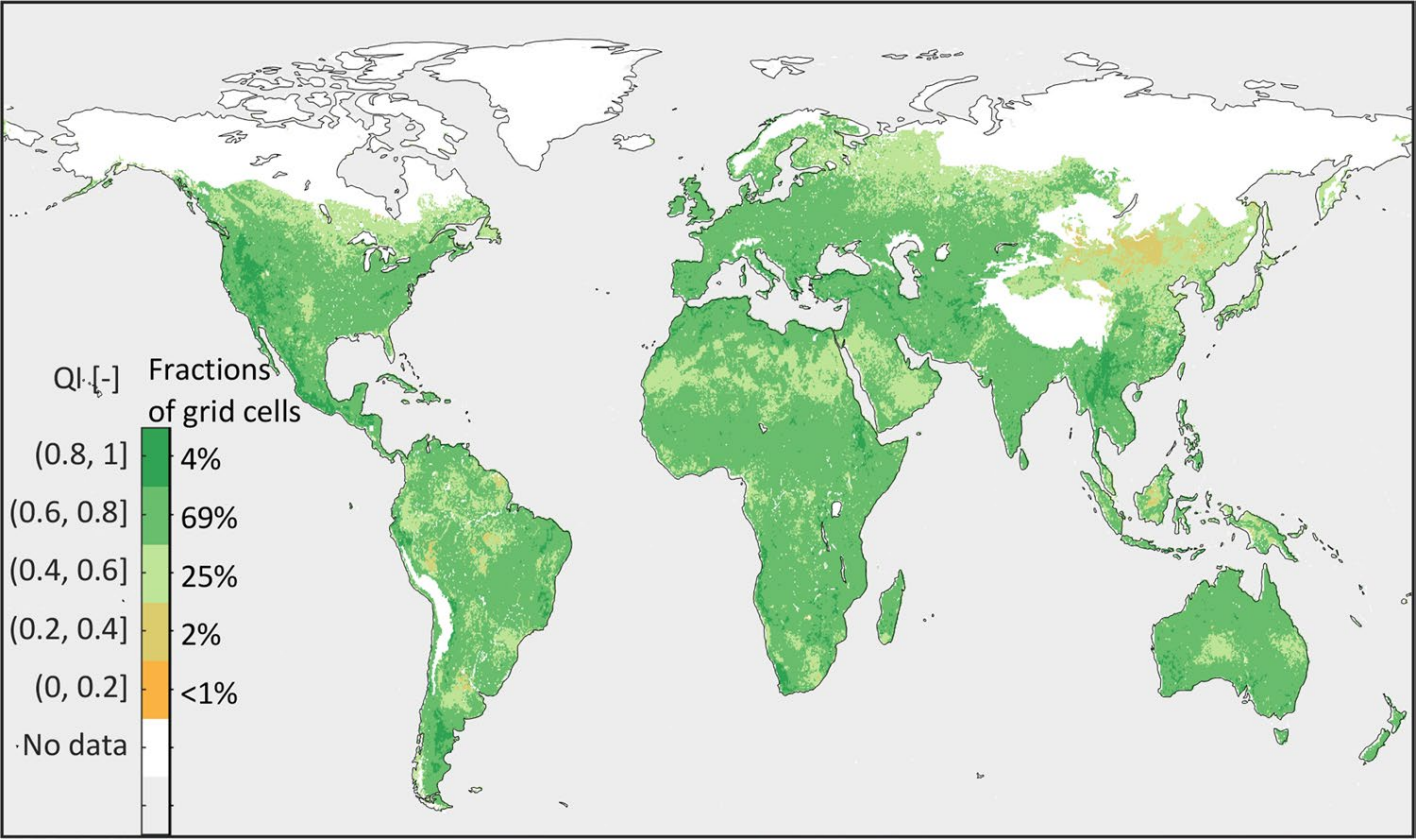
Data quality index *QI* combining the anomaly and temporal Pearson’s correlation between the long-term mean and long-term monthly mean of SOIL-WATERGRIDS estimates and the corresponding validation datasets calculated as prescribed in Eq. (11).

Equations (9) and (10) were combined to calculate the quality index $QI$ in each grid cell *i* as

11 $$QI\left(i\right)=\frac{Q{F}_{\Delta }\left(i\right)+Q{F}_{{\rm{R}}}\left(i\right)}{2}$$

where *QI* = 0 means “worse” and *QI* = 1 means “best” quality. Relative to the distributed variables, 69% of grid cells in SOIL-WATERGRIDS estimates have a *QI* between 0.6 and 0.8, and 4% have *QI* values between 0.8 and 1 (Fig. 5). The *QI* georeferenced data are distributed with the SOIL-WATERGRIDS distribution dataset in^49^.

### Known limitations

We acknowledge some limitations in this first version of SOIL-WATERGRIDS that may be addressed in future updates of the dataset.

The heterogeneity of source datasets in hydrometeorological conditions and land cover can result in some inconsistencies within and across interrelated data. For example, some inconsistencies observed between the potential and actual evapotranspiration from the CRU/TS and GLEAM datasets are quantified and highlighted in the Supplementary Information, but those inconsistencies may reduce in future releases of the original datasets or through implementation of numerical corrections across various datasets when used in combinations. Likewise, we have not explicitly accounted for the land cover change in the 45 year priod of assessment, and we used data relative to 2014. Incorporation of land cover change is challenging in modelling at these spatial and temporal scales because it implies changes in runoff but also rooting systems in soil, changes in atmospheric conditions such as temperature and relative humidity, albedo/reflectance, heat exchange fluxes, urbanization, and possibly many other aspects, part of which are not yet fully characterized. We do not have yet tools to fully account for land cover changes at the current stage of progression in our modelling capabilities; we presume that land changes are accounted for in some implicit way in the datasets we have used (e.g., precipitation, evapotranspiration) because those data are re-analyses based also on satellite observations. Hence, some biases observed in dry and humid regions may be caused by these uncertainties.

The calculation of surface runoff using the NRCS-CN method has advantages because it follows a uniform protocol to forecast the daily surface runoff that does not require additional calibration of parameters, hence it is relatively simple and stable in many applications^52^ but is also mostly empirical as compared to other mechanistic approaches such as TOPMODEL^53^. We introduced an element of process accounting via infiltration into soil using the Richards equations, but a better process description should explicitly describe the land surface runoff, stream flow, and groundwater flow. For these reasons, we conditioned the water balance in each grid cells of SOIL-WATERGRIDS for the surface runoff calculated using the NRCS-CN method and we tested against the GRUNv1 dataset whether this approach was able to represent surface processes. Nonetheless, the NRCS-CN method may lack in representation of snow, ice, and soil thawing, hence the inclusion of a water balance such as in our approach still limits the capability to describe soil water in regions that undergo freezing during part of the year, thus potentially affecting also downstream grid cells.

We also acknowledge that the computational domain we developed for SOIL-WATERGRIDS can describe the soil profile vertically down to 56 m depth, but deeper soil profiles may be needed to increase the capability to inform on global water assets relative to the lower water storage. Similarly, the spatial resolution across the latitude and longitude may be increased to reduce aliasing and use more effectively data that are distributed at high resolution such as SoilGridsv2 amongst others.

Overall comparison against available datasets provides a picture of where estimates in SOIL-WATERGRIDS stand as compared to existing ones. In particular, anomalies show the geographic distribution of differences and detailed time sequence analyses can provide also information on when anomalies may appear. It is worth to acknowledge that all datasets used for validation, including those based on observations such as the ESA/CCI, ISMN, and water table depth in^10^, may suffer from uncertainty and biases. Nonetheless, efforts in elaborating estimates of global scale water assets have an extraordinary importance in face of the current and expected planetary stresses on soil, water, and food security.

## Usage Notes

The SOIL-WATERGRIDS dataset and modelling distributions contain inputs to and outputs of our model. The former are mostly standard TXT formatted files readable in any freeware text editor (e.g., Textpad or similar), while the latter are standard NetCDF4 formatted files, which can be read in different coding languages (e.g., MATLAB, Python, Julia) or used within specialised licenced software (ArcGIS) and free software (e.g., QGIS, Panoply). The data release also contains a Technical Documentation reporting details on the SOIL-WATERGRIDS dataset and modelling distribution, including file organization, and editable custom codes written in MathWorks @Matlab 2019b.

## Supplementary information

Supplementary Information

## Data Availability

SOIL-WATERGRIDS has been generated using the BRTSim (BioReactive Transport Simulator) computational solver version v4.1a (2020). The BRTSim software is multiplatform and can be deployed on Microsoft, Unix/Linux/Ubuntu, and Mac operating systems. The full BRTSim package, inclusive of executables, examples, basic post-processing scripts, and User Manual and Technical Guide are available for download at the BRTSim home page https://sites.google.com/site/thebrtsimproject/home under the CC BY4.0 license. Seeding datasets used in our modelling are not distributed here because they are publicly accessible from the links reported in Online-only Table 1. BRTSim input files required to produce the full-size global-scale simulation of SOIL-WATERGRIDS are distributed in this data release and are available at the Zenodo repository^49^ (about 50 GB). The BRTSim modelling distribution package for SOIL-WATERGRIDS includes 7 continental regions (Africa, Asia insular, Asia, Europe, North America, South America, Oceania) organized in compressed folders that include the BRTSim executable and license file, bash files (BRTSim_v41a_GLNXA64_R2019b, run_BRTSim_v41a_GLNXA64_R2019b.sh, license.txt), the Param_*.inp files to instruct BRTSim to run each grid cells of the computational domain, and the tables of boundary conditions (Table_*.txt, WB_*.txt, and RNF_*.txt) organized by continental regions. The license file is valid until the end of 2021 and a new license can be obtained with no charge from the BRTSim home page after expiry. The distributed package allows to model about 168,000 grid cells globally. Raw outputs of the full-scale model run occupy about 10TB (uncompressed) and is not distributed. Full details on the BRTSim modelling including data structure, file naming, supported operating system, modelling launching and workflow, and accompanying scripts are available in the SOIL-WATERGRIDS Technical Documentation^49^.

## Online-only Table

**Online-only Table 1:** Global datasets used in this work.

Variables	Data type	Data Source	Description	Format
Soil texture, bulk density	S	SoilGrids2.0^29^	Sand/silt/clay fractions at six depths from land surface to 1.6 m. Globally gridded at 5 arc-min resolution (about 10 km at the equator). Last accessed on August 2020 at http://maps.isric.org/	.TIF
Soil porosity	S	SoilGrids1.0^30^	Expressed in [%] at seven depths from the surface to 2 m depth. Globally gridded at 5 arc-min resolution (about 10 km at the equator). Last accessed in 2018 at ftp://ftp.soilgrids.org/data/recent/	.TIF
Soil hydraulics parameters	S	^32^	Profiles at eight depths from the surface to 2.3 m depth. Air-entry suction expressed in [cm], hydraulic conductivity at saturation expressed in [cm/day], and pore volume distribution index [-]. Globally gridded at 30 arc-sec resolution (about 1 km at the equator). Last accessed in October 2019 at http://globalchange.bnu.edu.cn/research	.NC
Soil residual water content	S	^47^	Expressed in [cm^3^ cm^−3^]. Globally gridded at 30 arc-sec resolution (about 1 km at the equator). Last accessed in November 2019 at https://dataverse.harvard.edu/dataset.xhtml?persistentId=doi:10.7910/DVN/UI5LCE	.TIF
Soil thickness	S	ORNL/DAAC^78^	Expressed in [m]. Globally gridded at 30 arc-sec resolution (about 1 km at the equator). Last accessed in September 2018 at https://daac.ornl.gov/cgi-bin/dsviewer.pl?ds_id=1304	.TIF
Water table depth	WTD	^10^	Equilibrium and long-term monthly averages expressed in [m] from land surface. Globally gridded at 30 arc-sec (about 1 km at the equator). Years covered: 2003 to 2013. Last accessed in September 2020 at https://aquaknow.jrc.ec.europa.eu/content/global-patterns-groundwater-table-depth-wtd	.NC
Soil moisture	SM	NOAH/GLDAS^19,79^	Expressed in [kg m^−2^]. Globally gridded at 0.25 degree resolution (about 28 km at the equator) and monthly frequency. Years covered: 1948 to 2014. Last accessed in October 2020 at https://disc.gsfc.nasa.gov/	.NC
Soil moisture	SM	GLEAM^25^	Expressed in [m^3^ m^−3^]. Globally gridded at 0.25 degree resolution (about 28 km at the equator) and monthly frequency. Years covered: 1980 to 2018. Last accessed in October 2020 at https://www.gleam.eu/#downloads	
Soil moisture	SM	ESA/CCI^21–23^	Expressed in [m^3^ m^−3^]. Globally gridded at 0.25 degrees (about 28 km at the equator) and monthly frequency. Years covered: 1979 to 2019. Data version:ESA CCI Soil Moisture Product New Version Release (v04.7). Last accessed in June 2020 at https://www.esa-soilmoisture-cci.org	.NC
Soil moisture	SM	ISMN network^27^	Long-term monthly averages expressed in [m^3^ m^−3^]. Single location data from different networks combined in a global grid at 0.25 degree resolution (about 28 km at the equator). Last accessed in August 2020 at https://www.geo.tuwien.ac.at/insitu/data_viewer/	.csv
Land surface temperature	HM	NOAA/NCEI^80^	Expressed in [◦C]. Globally gridded at 15 arc-min resolution (about 28 km at the equator). Year covered: 2001. Last accessed in November 2018 at ftp.ngdc.noaa.gov	.NC
Rainfall, atmospheric temperature, potential evapotranspiration	HM	CRU/TS^41^	Daily rainfall and monthly potential evapotranspiration expressed in [mm/day] and [mm/month], respectively, and temperature expressed in [°C]. Globally gridded at 0.5 degree resolution (about 55 km at the equator) and daily frequency. Years covered: 1970 to 2018. Last accessed in April 2020 at https://crudata.uea.ac.uk/cru/data/hrg/	.NC
Actual evapotranspiration	HM	GLEAM^24,25^	Expressed in [mm/month]. Globally gridded at 0.25-degree resolution (about 28 km at the equator) and monthly frequency. Years covered: 1981 to 2018. Last accessed in October 2020 at https://www.gleam.eu/#downloads	.NC
Total Runoff	HM	GRUNv1^28^	Expressed in [mm/monthly]. Globally gridded at 0.5 degrees resolution (about 55 km at the equator) and monthly frequency. Years covered: 1902 to 2014. Last accessed in November 2020 at https://www.research-collection.ethz.ch/handle/20.500.11850/324386?show=full	.NC4
Land cover	L	MODIS/IGBP 2019^34,35^	Land cover classification of the IGBP. Globally gridded at 0.05 degree resolution (about 5 km at the equator). Years covered: 2001 to 2018. Last accessed in August 2019 at https://lpdaac.usgs.gov/products/mcd12q1v006/	.HDF
Root density profile for crops	L	^36^	Maximum and mean root depth in agricultural land expressed in [m]. Globally gridded at 5 arc-min resolution (about 10 km at the equator). The maps are relative to year 2000. Calculated as described in publication.	.TIF
Root density profile for native ecosystems	L	^37^	Maximum root depth in [m] for different ecosystems. Available from publication.	Table
Crop water security	L	NASA/LPDAAC^39^	Expressed by classification ranking. Globally gridded at 5-arc-min resolution (about 10 km at the equator). Last accessed in January 2019 at https://lpdaac.usgs.gov/dataset_discovery/measures/measures_products_table/gfsad1kcm_v001/	.TIF
Crop calendars	L	^40^	Planting and harvesting time expressed in day of the year for 25 crops. Globally gridded at 5 arc-min resolution (about 10 km at the equator). Last accessed in November 2018 at https://nelson.wisc.edu/sage/data-and-models/crop-calendar-dataset/index.php	.NC
Digital elevation map	L	ETOPO5^45^	Expressed in [m]. Globally gridded at a 5 arc-minute resolution grid (about 10 km at the equator). Last accessed in August 2020 at https://www.eea.europa.eu/data-and-maps/data/world-digital-elevation-model-etopo5.	.TIF
Permafrost	L	NASA/NEO^46^	Expressed in % area fraction. Globally gridded at 0.1 degree resolution (about 1.1 km at the equator). Last accessed in November 2018 at https://nsidc.org/data/ggd318	. SPH

Acronyms in column “Data type” stand for: S, soil properties; WTD, water table depth; SM, soil moisture; L, land cover and use; HM, hydrometeorology.
