# Supplementary material for: SOIL-WATERGRIDS, mapping dynamic changes in soil moisture and depth of water table from 1970 to 2014
Source: Sci Data. 2021 Oct 6;8:263. doi: 10.1038/s41597-021-01032-4 (PMC8494894; doi:10.1038/s41597-021-01032-4)

**Supplementary Information for**

**SOIL-WATERGRIDS, mapping dynamic changes in soil moisture and depth of water table from 1970 to 2014**

### Authors

Magda Guglielmo^1^, Fiona H. M. Tang^1^ , Chiara Pasut^1^, Federico Maggi^1^

^1^Laboratory for Advanced Environmental Engineering Research, School of Civil Engineering, The University of Sydney, Bld. J05, 2006 Sydney, NSW, Australia.

Corresponding author(s): Magda Guglielmo ([magda.guglielmo@sydney.edu.au](mailto:magda.guglielmo@sydney.edu.au)), Fiona Tang ([fiona.tang@sydney.edu.au](mailto:fiona.tang@sydney.edu.au)), Chiara Pasut ([c.pasut@sydney.edu.au](mailto:c.paust@sydney.edu.au)), Federico Maggi ([federico.maggi@sydney.edu.au](mailto:federico.maggi@sydney.edu.au))

### Content

This Supplementary Information document contains graphical material in support to the material presented in the main paper (Guglielmo et al., 2021). The list of contents is introduce below:

**Figure S1.** Overall data type and contruction workflow of SOIL-WATERGRIDS, pag 3.

**Figure S2.** Phycnophilactic quality of data harmonization, pag 4.

**Figure S3.** Data consistency, pag 4.

**Figure S4.** Reconstrunstruction quality of missing actual evapotranspiration, pag 5.

**Figure S5.** Computational domain (ROI) of SOIL-WATERGRIDS, pag 5.

**Figure S6.** Quality of corrections of soil hydraulic propoerties, pag 6.

**Figure S7.** Geographic distribution of dry and humid regions, pag 6.

**Figure S8.** Anomaly maps of volumetric soil water content, pag 7.

**Figure S9.** Seasonality correlation map between SOIL-WATERGRIDS and other data sets, pag 8.

**Figure S10.** Long-term mean maps of volumetric soil water content, pag 8.

**Figure S11.** Anomaly maps of water table depth, pag 9.

**Figure S12.** Number of water tables and long-term mean map of water deble depth, pag 9.

**Figure S13**. Map of ponding and wetlands, pag 10.

**Figure S14.** Upper bound and correction introduced with water balance, pag 10.

**References to datasets**

GLEAM, Miralles, et al., (2011); Martens, et al., (2017).

ESA/CCI, Gruber et al., (2017); Gruber et al., (2019); Dorigo et al, (2017).

ISMN, Dorigo et al., (2011).

NOAH/GLDAS, Rodell et al., (2004); Beaudoing and Rodell (2019).

SWAMPS, Poulter et al (2017)

GRUNv1, Ghiggi et al., 2019.

**References**

Beaudoing, H. & Rodell, M. NASA/GSFC/HSL (2019), GLDAS Noah Land Surface Model L4 monthly 0.25 x 0.25 degree V2.0, *Greenbelt, Maryland, USA, Goddard Earth Sciences Data and Information Services Center (GES DISC)*, [10.5067/9SQ1B3ZXP2C5](https://doi.org/10.5067/9SQ1B3ZXP2C5)

Dai, Y. *et al*. A Global High-Resolution Data Set of Soil Hydraulic and Thermal Properties for Land Surface Modeling. *J. Adv. Model*, **11**, 2996-3023 (2019).

Dorigo, W. *et al*. ESA CCI Soil Moisture for improved Earth system understanding: State-of-the art and future directions. Remote. Sens. Environ., 203, 185-215 (2017).

Dorigo, W. A. *et al.* The International Soil Moisture Network: a data hosting facility for global in situ soil moisture measurements*. Hydrol Earth Syst Sci*, **15**, 1675-1698, (2011).

Ghiggi, G., Humphrey, V., Seneviratne, S. I., & Gudmundsson, L. (2019). GRUN: an observation-based global gridded runoff dataset from 1902 to 2014, Earth Syst. Sci. Data, 11, 1655–1674.

Guglielmo, M., Tang, F. H. M., Pasut, C., Maggi, F., (2021). Soil-WATERGRIDS, mapping dynamic changes in soil moisture and depth of water table from 1970 to 2014. Scientific Data.

Gruber, A., Dorigo, W. A., Crow, W. & Wagner, W. Triple Collocation-Based Merging of Satellite Soil Moisture Retrievals. *IEEE Trans. Geosci. Remote Sen.*, **55**, 6780-6792 (2017).

Gruber, A., Scanlon, T., Schalie, R., Wagner, W.,& Dorigo, W. Evolution of the ESA CCI Soil Moisture climate data records and their underlying merging methodology. *Earth Syst. Sci. Data*, **11**, 717-739 (2019).

Poulter, B.; Bousquet, P.; Canadell, J.G.; Ciais, P.; Peregon, A.; Saunois, M.; Arora, V.K.; Beerling, D.J.; Brovkin, V.; Jones, C.D.; et al. Global wetland contribution to 2000–2012 atmospheric methane growth rate dynamics. Environ. Res. Lett. 2017, 12, 094013.

Rodell, M. *et al*. The Global Land Data Assimilation System. *Bull. Am. Meteorol. Soc.*, **85**, 381-394 (2004).

**Figure S1**. Conceptual workflow adopted to generate SOIL-WATERGRIDS. Acronyms in seeding data redirect to datasets in on-line only Table 1 in the main paper (Guglielmo et al., 2021): HM, hydrometeorological variables; S, soil properties; L, land use and cover characteristics; WTD, water table depth; and SM, soil moisture.


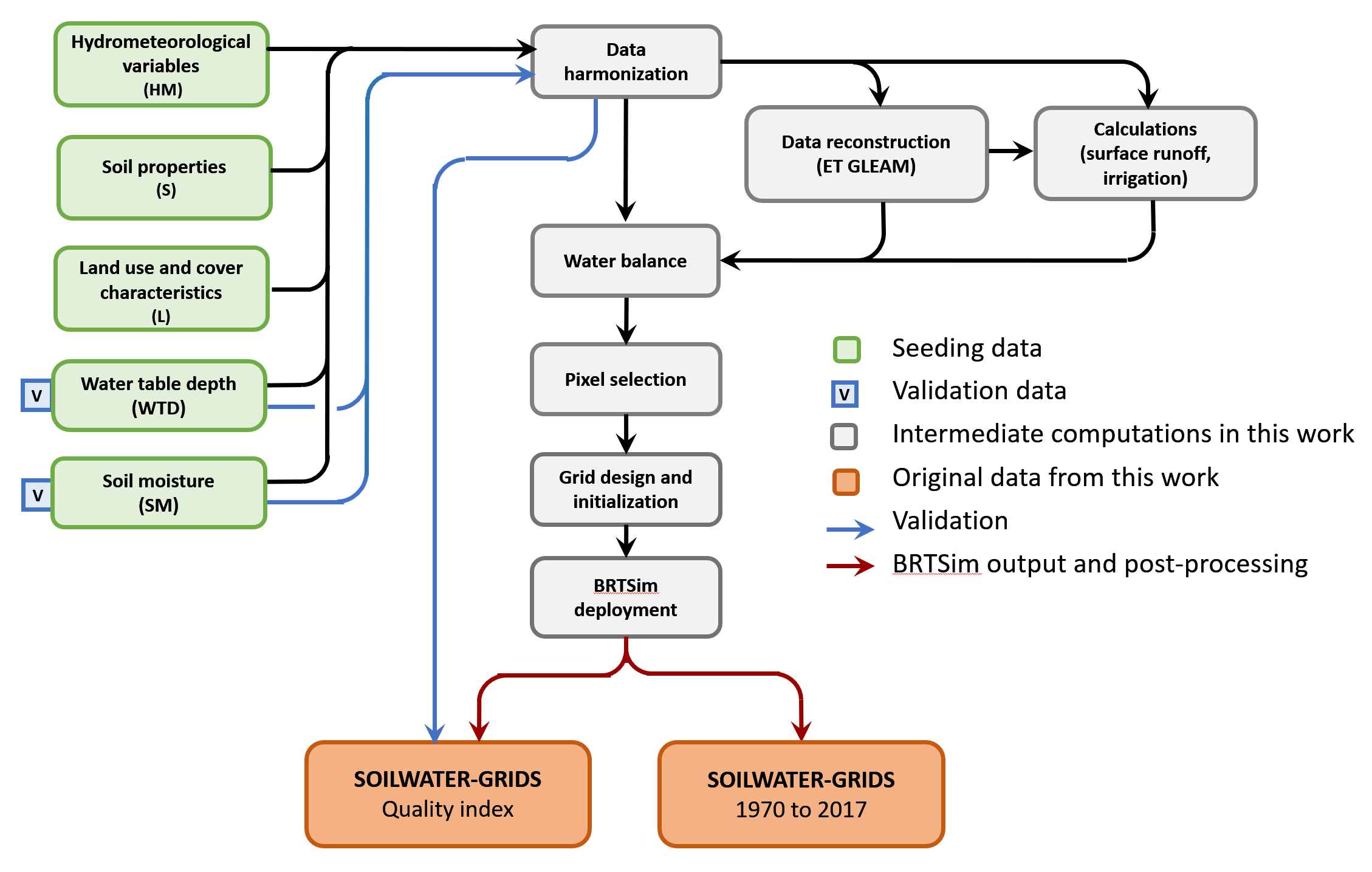


**Figure S1**. Geographic distribution of dry and humid regions in SOIL-WATERGRIDS identified by grid cells where $\theta$ in the top soil is below 0.8×$\theta_{FC}$ and above $\theta_{FC}$ , respectively, for 75% of the time within the 45 years of assessment from 1970 to 2014. $\theta_{FC}$ is the volumetric water content at field capacity, which corresponds to *ψ* = -33 kPa suction.


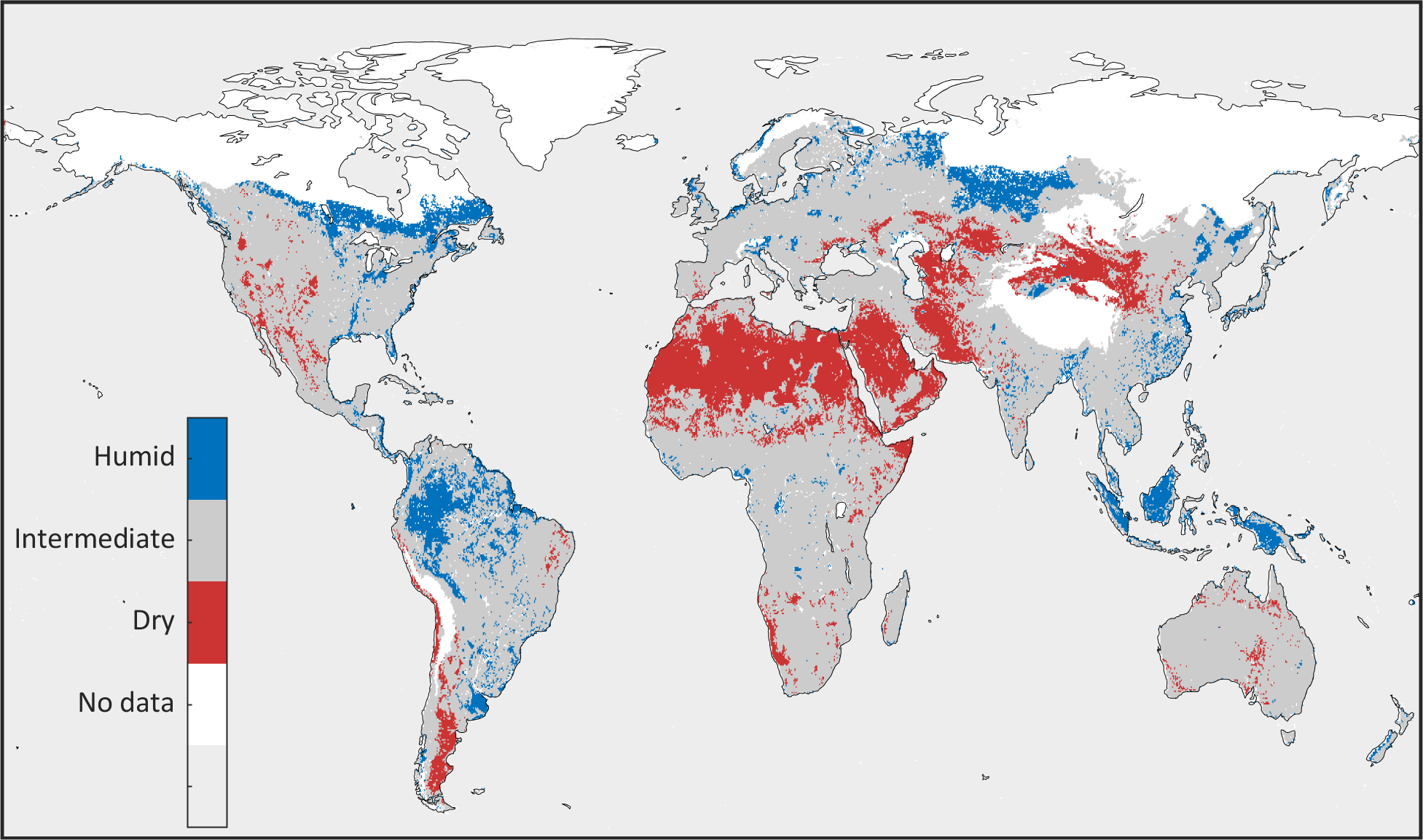

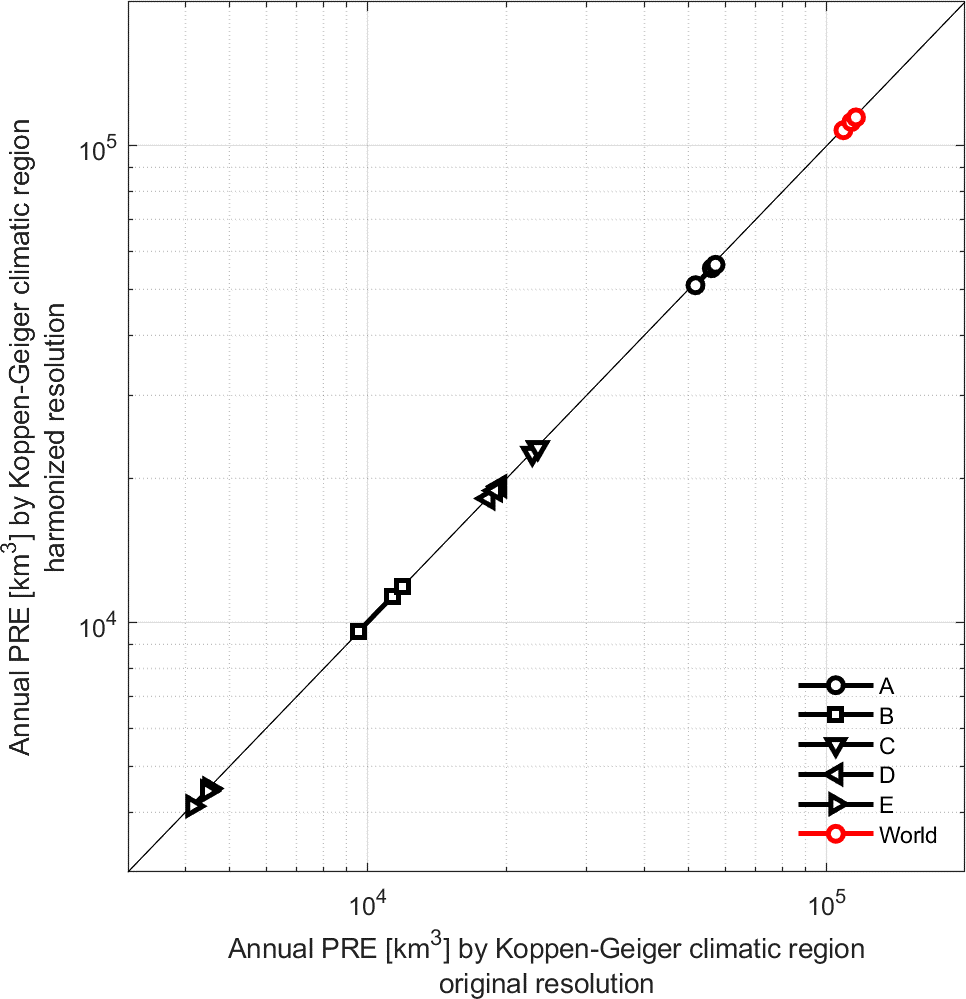

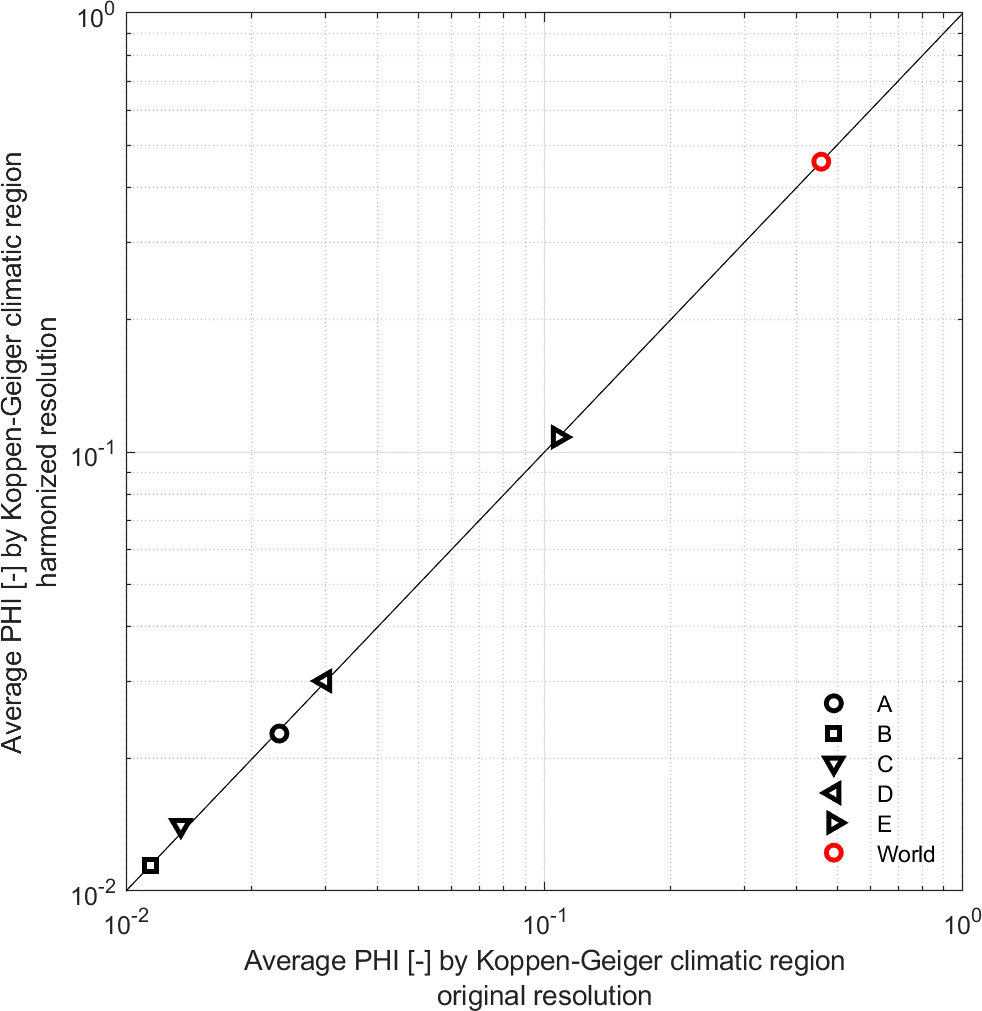


**Figure S2.** (a) Annual cumulative precipitation (PRE) in years 1990, 2000, and 2010 in the level-1 Koppen-Geiger climate region aggregation of harmonized as compared to original resolution data of the CRU/TS. (b) average soil porosity (PHI) in the top soil (0 to 30 cm) in the level-1 Koppen-Geiger climate region aggregation of harmonized as compared to original resolution data in SoilGrids v1. Level-1 Koppen-Geiger climate regions are: A, equatorial; B, arid; C, warm temperate; D, boreal; E, polar. Insets show the percent error relative to the original resolution of the data sets.


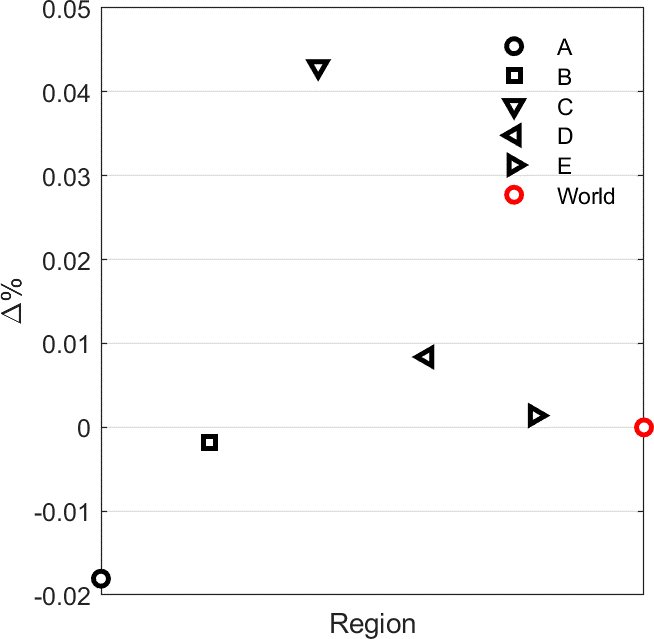

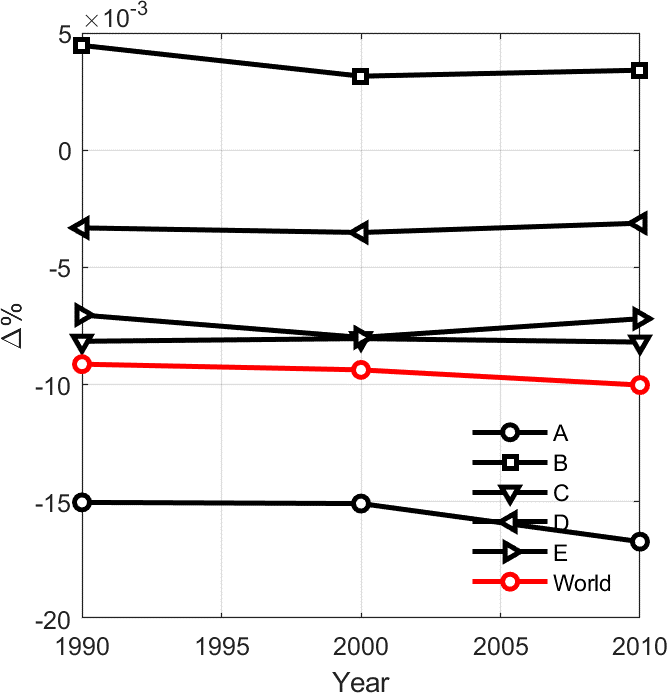


**Figure S3**. Data consistency between annual potential evapotranspiration *PET* of the CRU/TS dataset and actual evapotranspiration *ETA* of the GLEAM dataset over the computational domain of SOIL-WATERGRIDS. Data consistency is shown as the percent grid cells in the computational domain that satisfy the relationship *ETA* <= 1.20 *PET*, while inconsistencies exist when *ETA* > 1.20 *PET*.


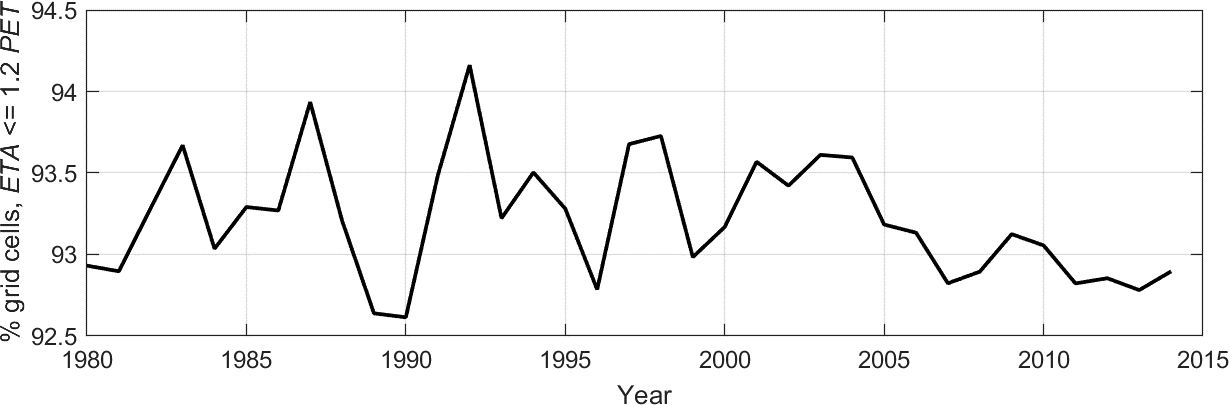

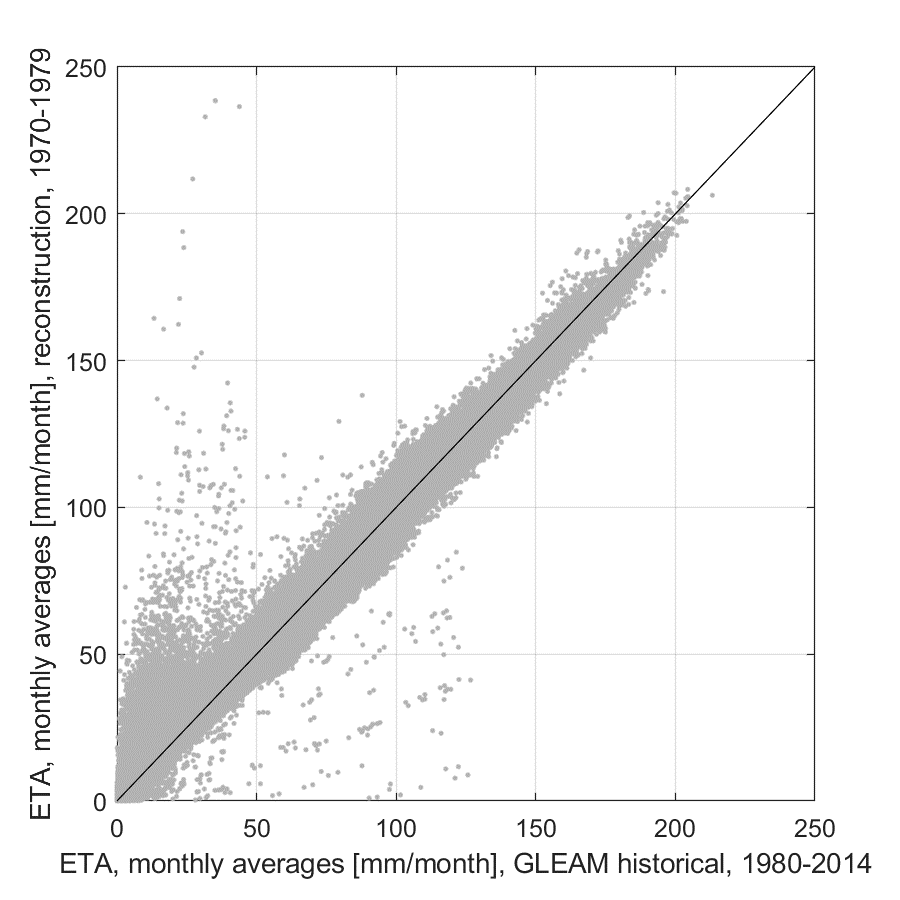


**Figure S4** Reconstructed against historical long-term monthly mean actual evapotranspiration from GLEAM calculated as in Eq. (1) in the main paper (Guglielmo et al., 2021).

**Figure S5**. Region of interest of SOIL-WATERGRIDS.


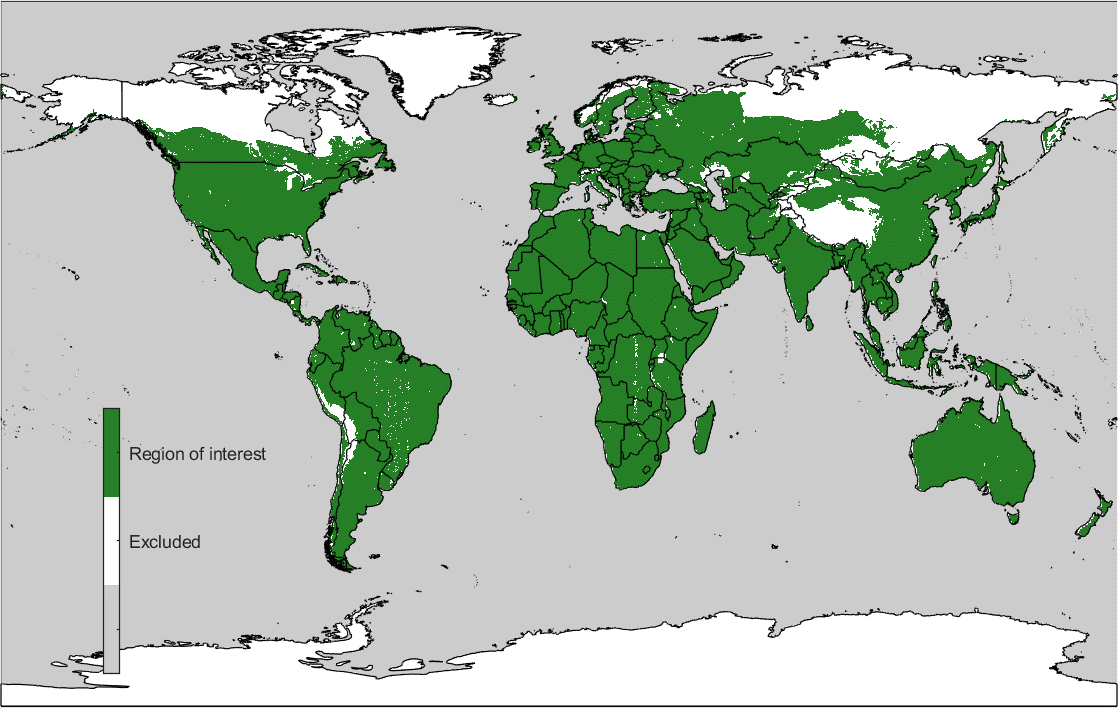


**Figure S7**. Geographic distribution of dry and humid regions in SOIL-WATERGRIDS identified by grid cells where $\theta$ in the top soil is below 0.8×$\theta_{FC}$ and above $\theta_{FC}$ , respectively, for 75% of the time within the 45 years of assessment from 1970 to 2014. $\theta_{FC}$ is the volumetric water content at field capacity, which corresponds to *ψ* = -33 kPa suction.


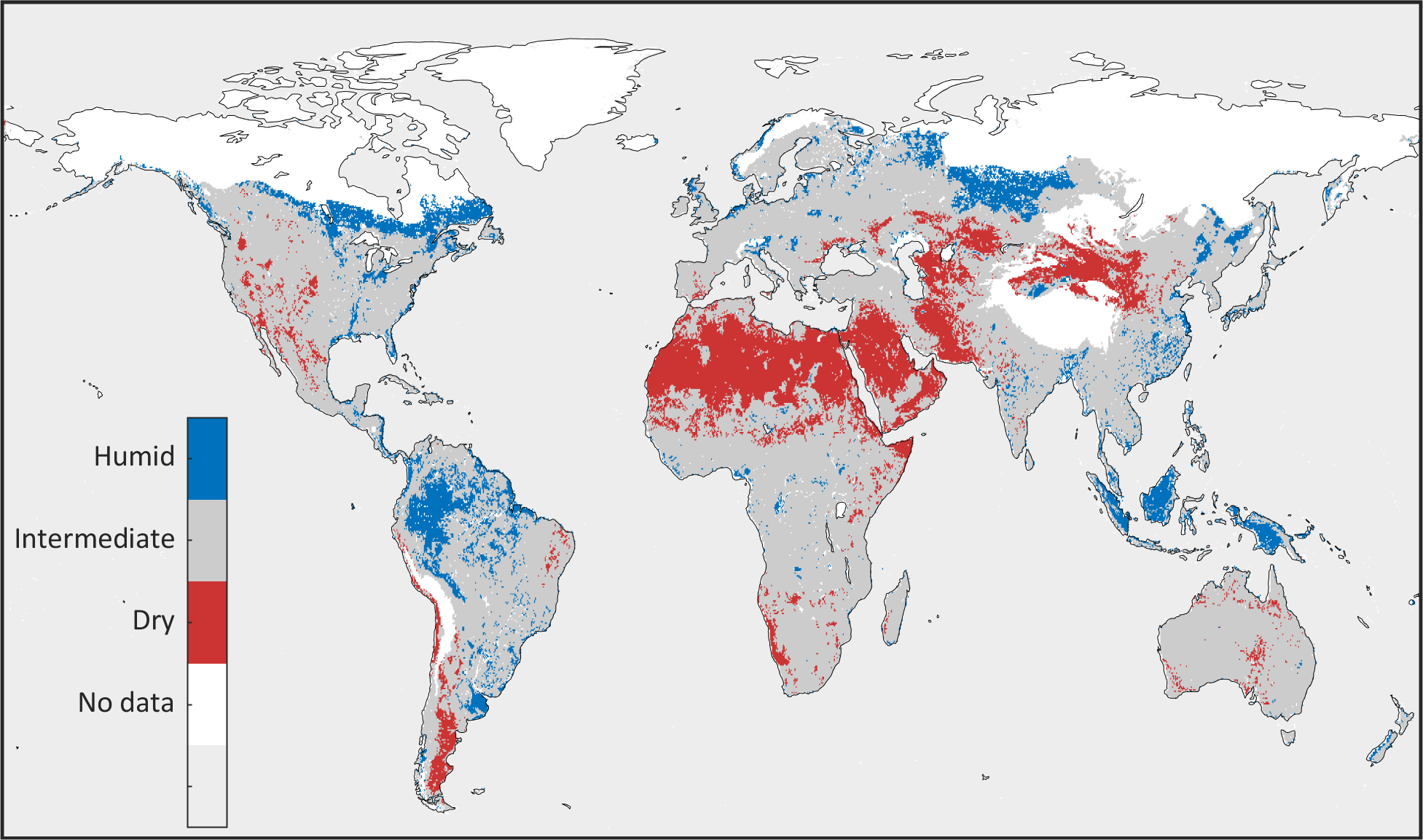


**Figure S6**. Frequency distribution of (first column) pore volume distribution index b, (second column) air-entry suction $\psi_{s}$ , and (third column) soil porosity$\phi$ at three depths within the root zone of SOIL-WATERGRIDS as compared to original sources in Dai et al., (2019). Globally gridded hydraulic parameters are distributed in file SOIL-WATERGRIDS_ext.nc (see Section “Data Records” in the main paper (Guglielmo et al., 2021).


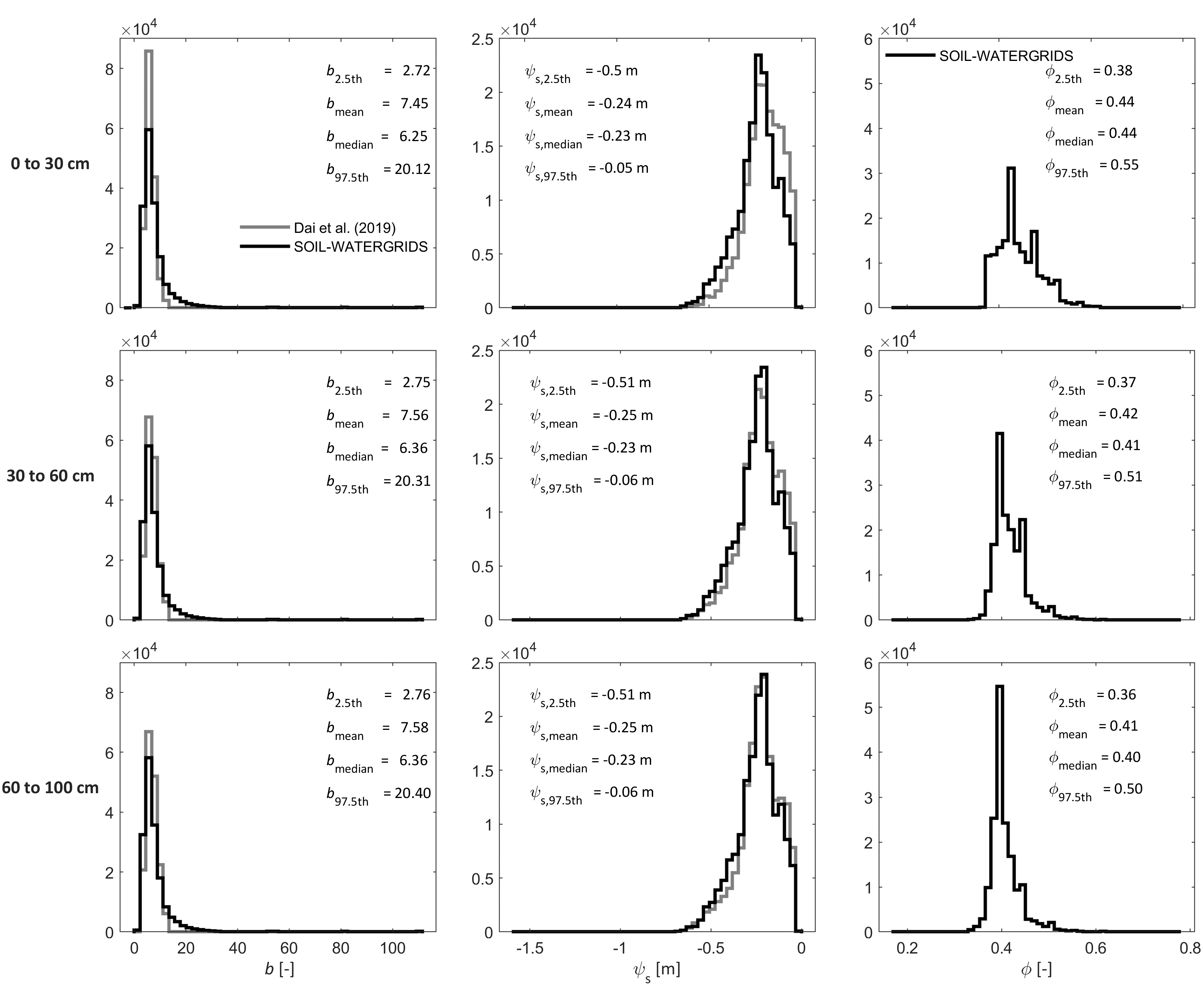

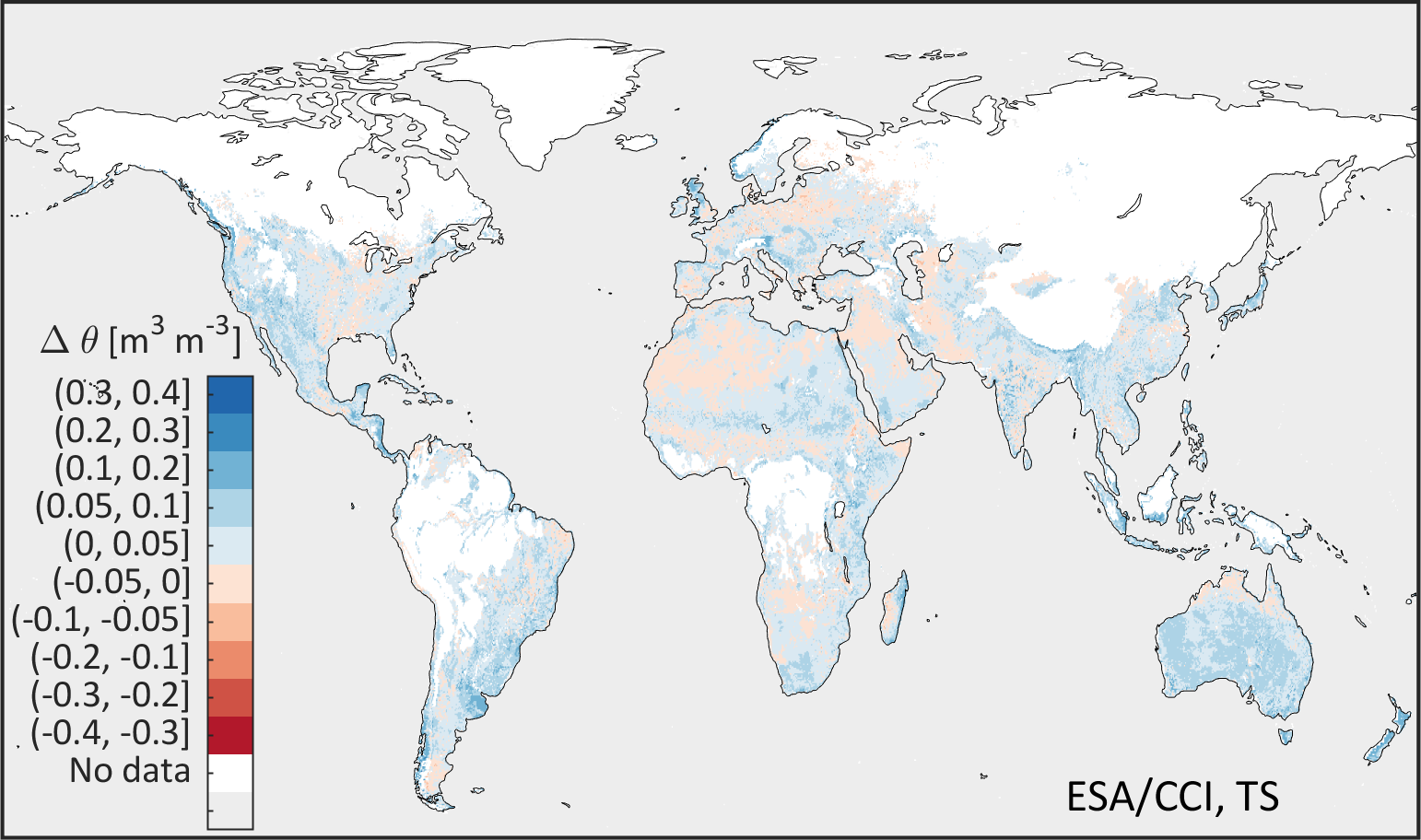

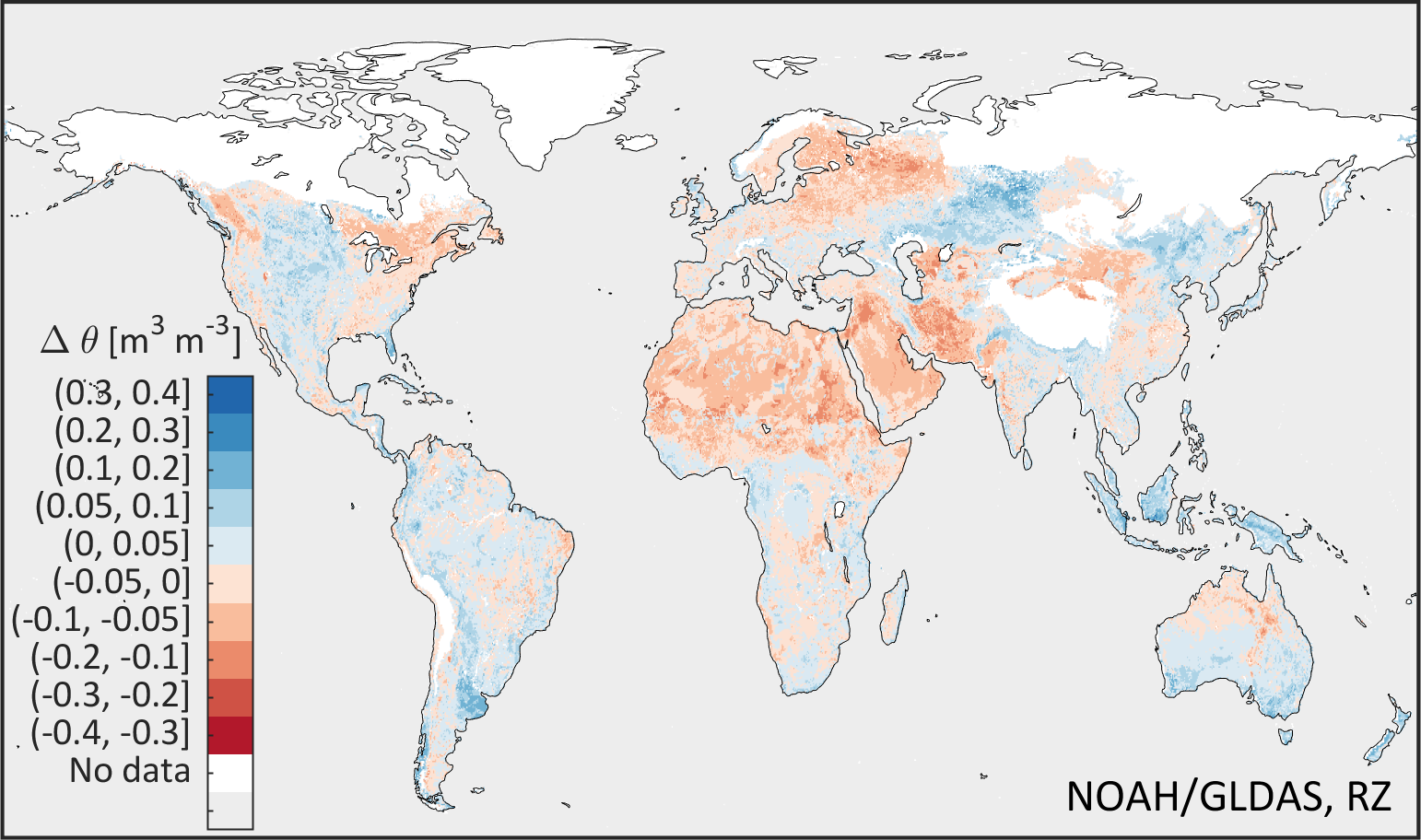

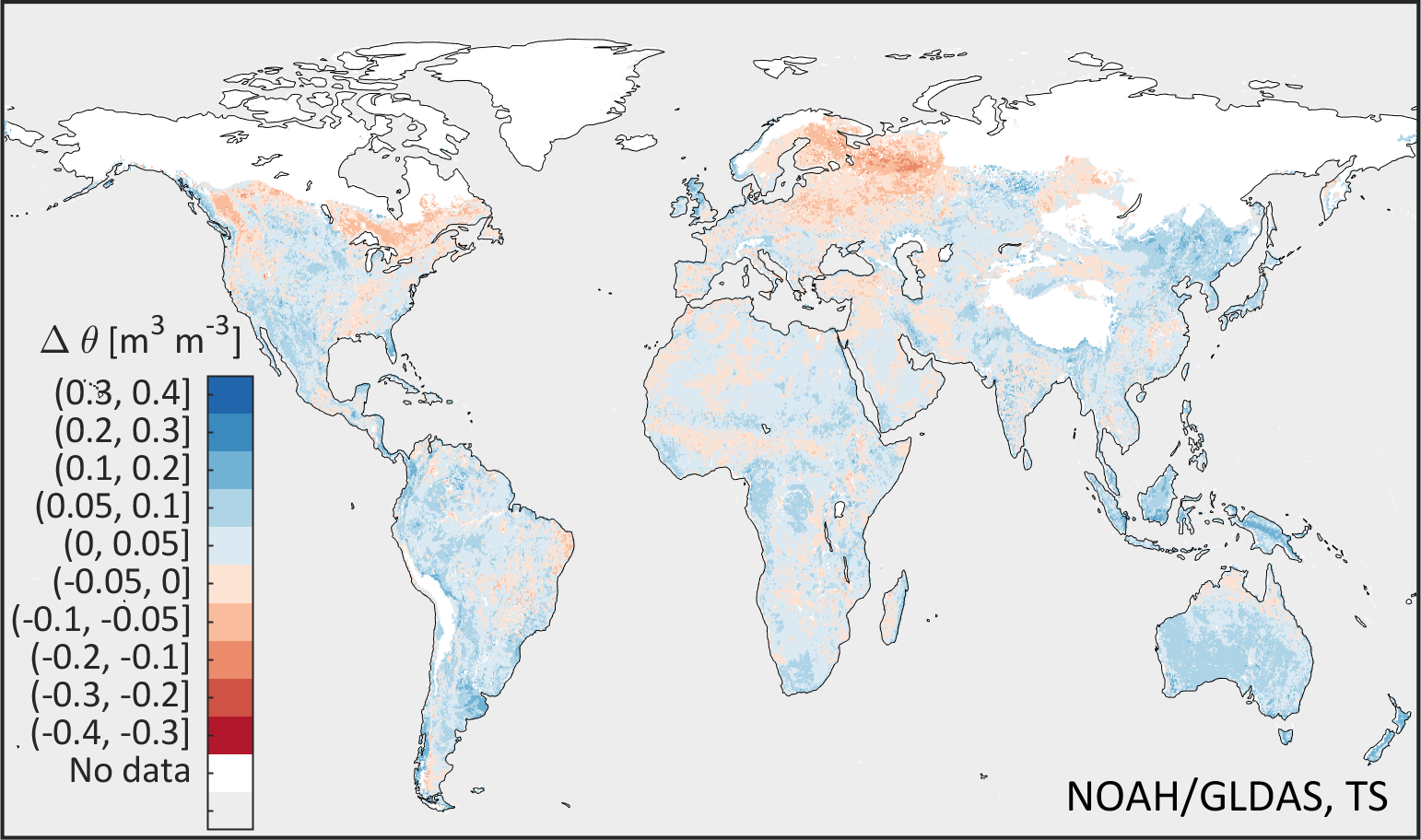

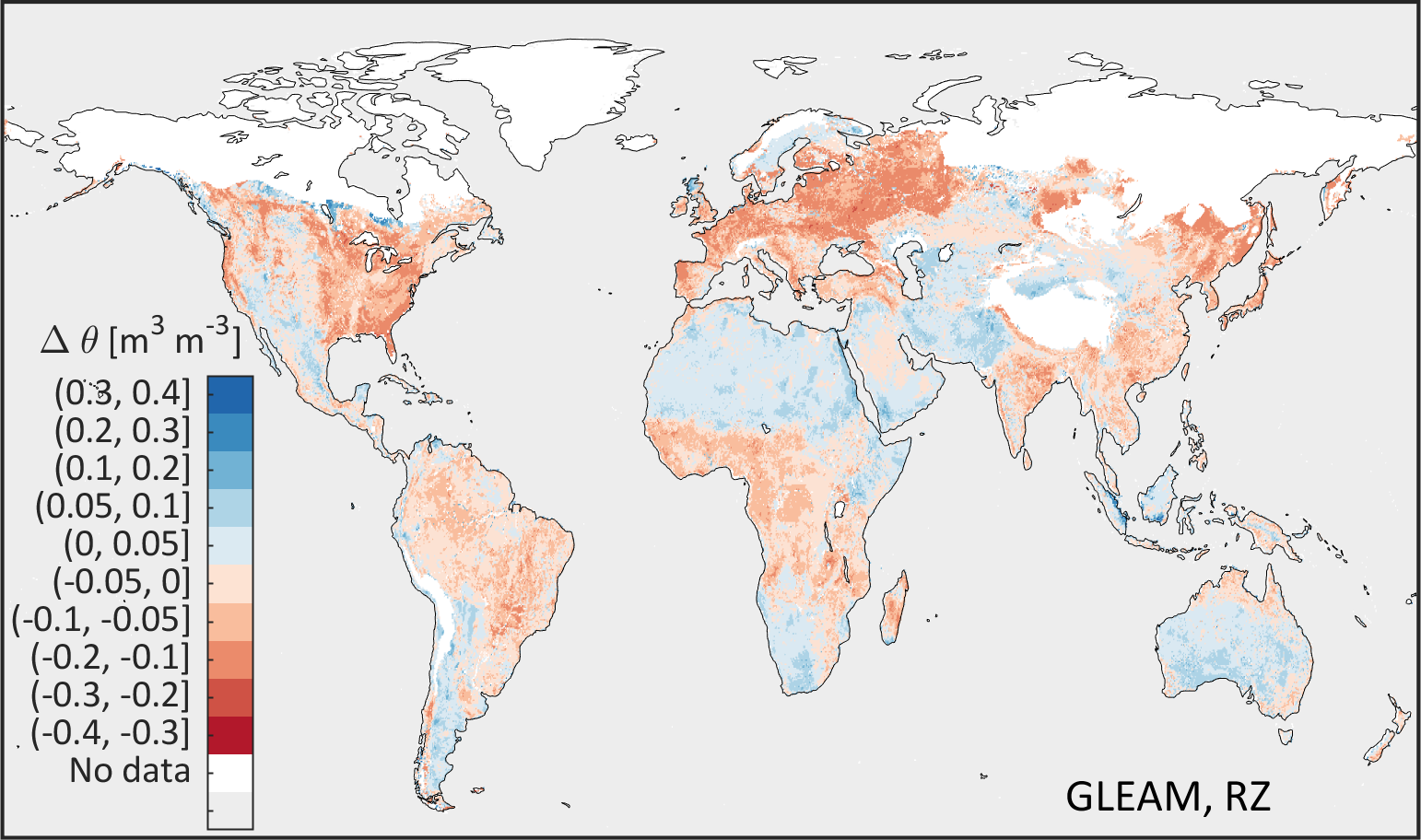

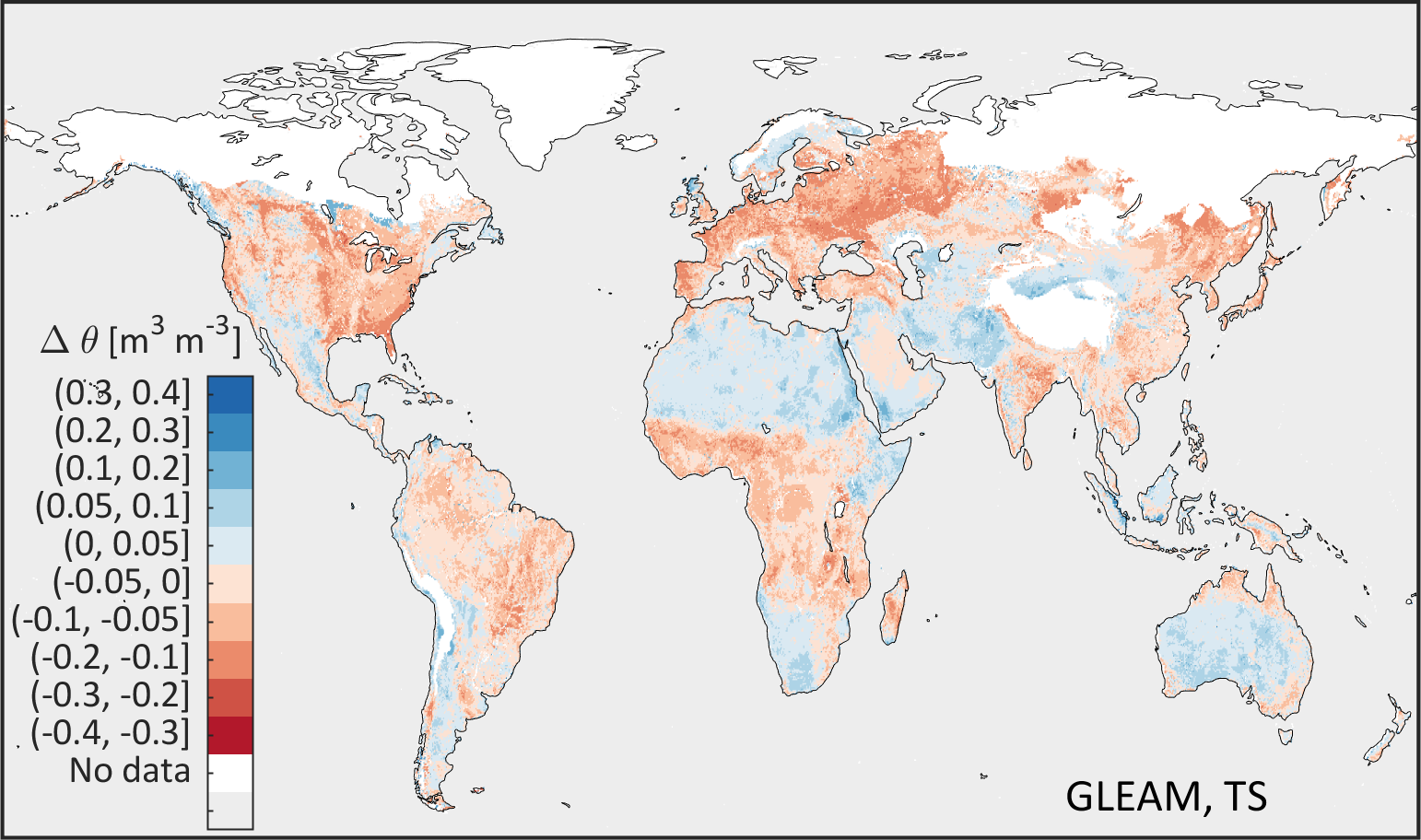

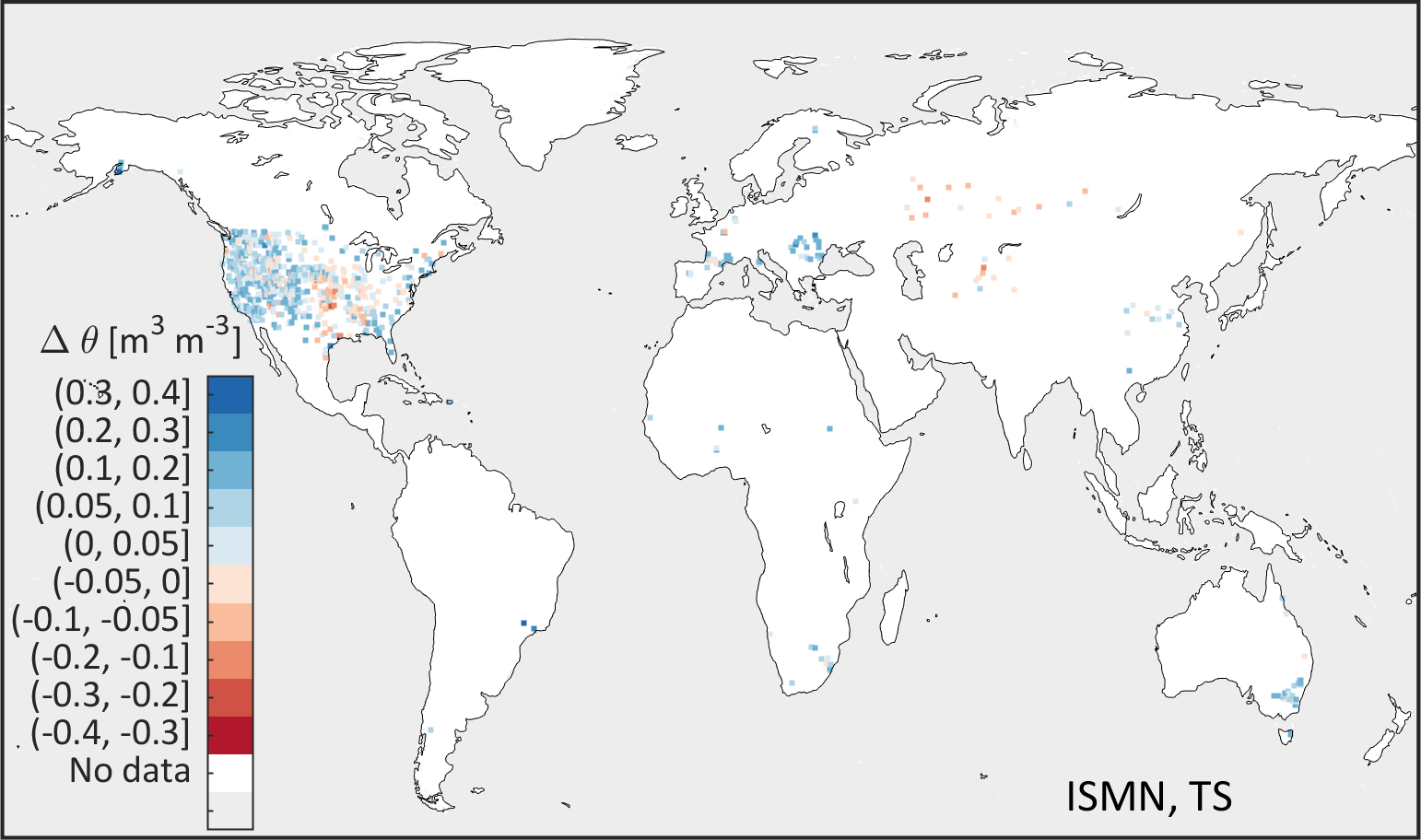


(a) (b)

(c) (d)

(e) (f)

**Figure S8**. Geographic distribution of the anomaly Δ$\theta$ in volumetric water content $\theta$ in the top soil (TS, 0 to 30 cm, panels a, b, c and d) and the root zone (RZ, 0 to 100 cm, panels e and f) against datasets from ESA/CCI, ISMN, NOAH/GLDAS, and GLEAM. The long-term mean values of $\theta$ in TS and RZ of SOIL-WATERGRIDS are calculated over the assessment period 1970-2014.

**Figure S10.** (a) and (b) long-term mean volumetric soil water content $\theta$ in the top soil (TS, 0 to 30 cm) and the root zone (RZ, 0 to 100 cm), respectively.The mean is calculated over the period of assessment from 1970 to 2014.


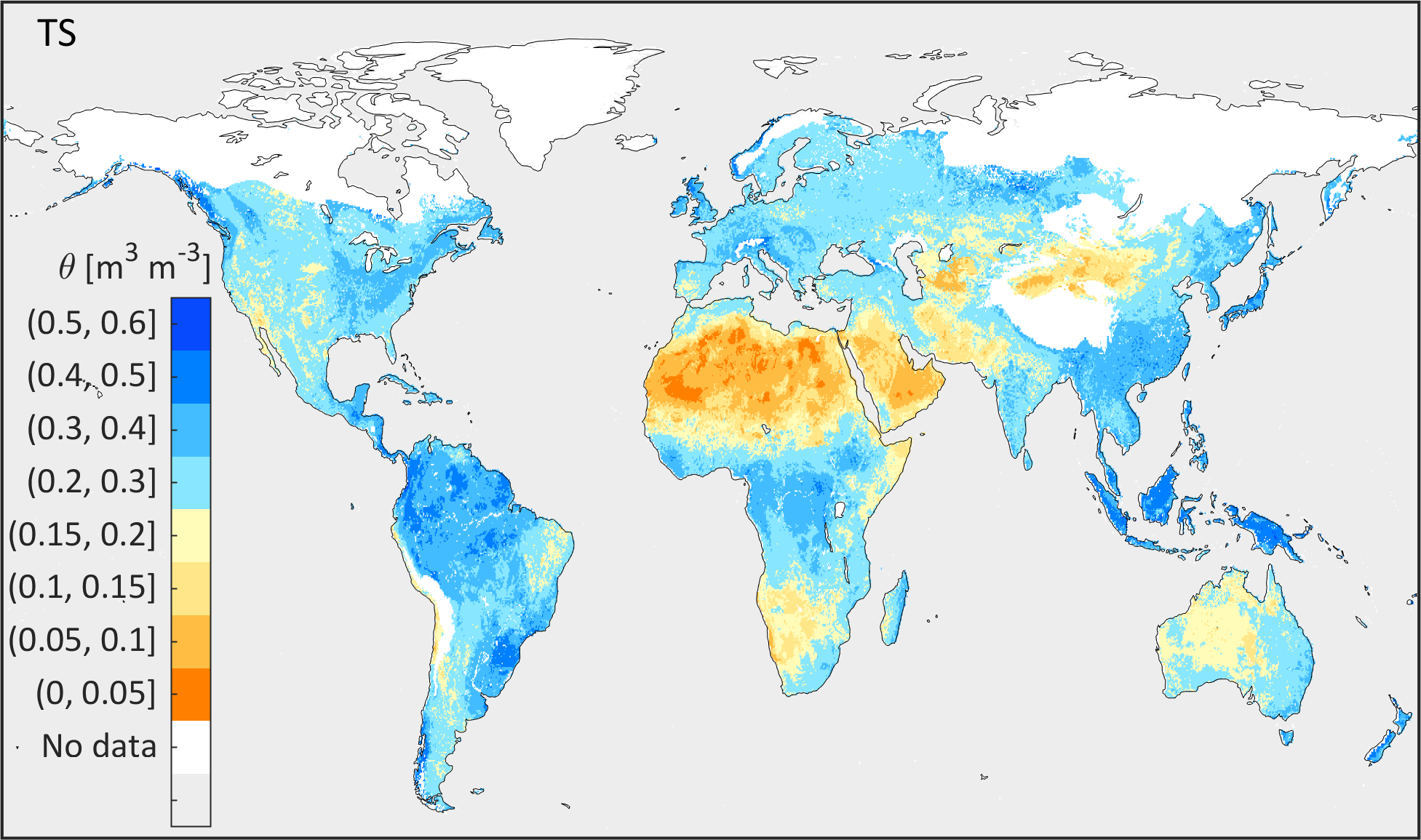

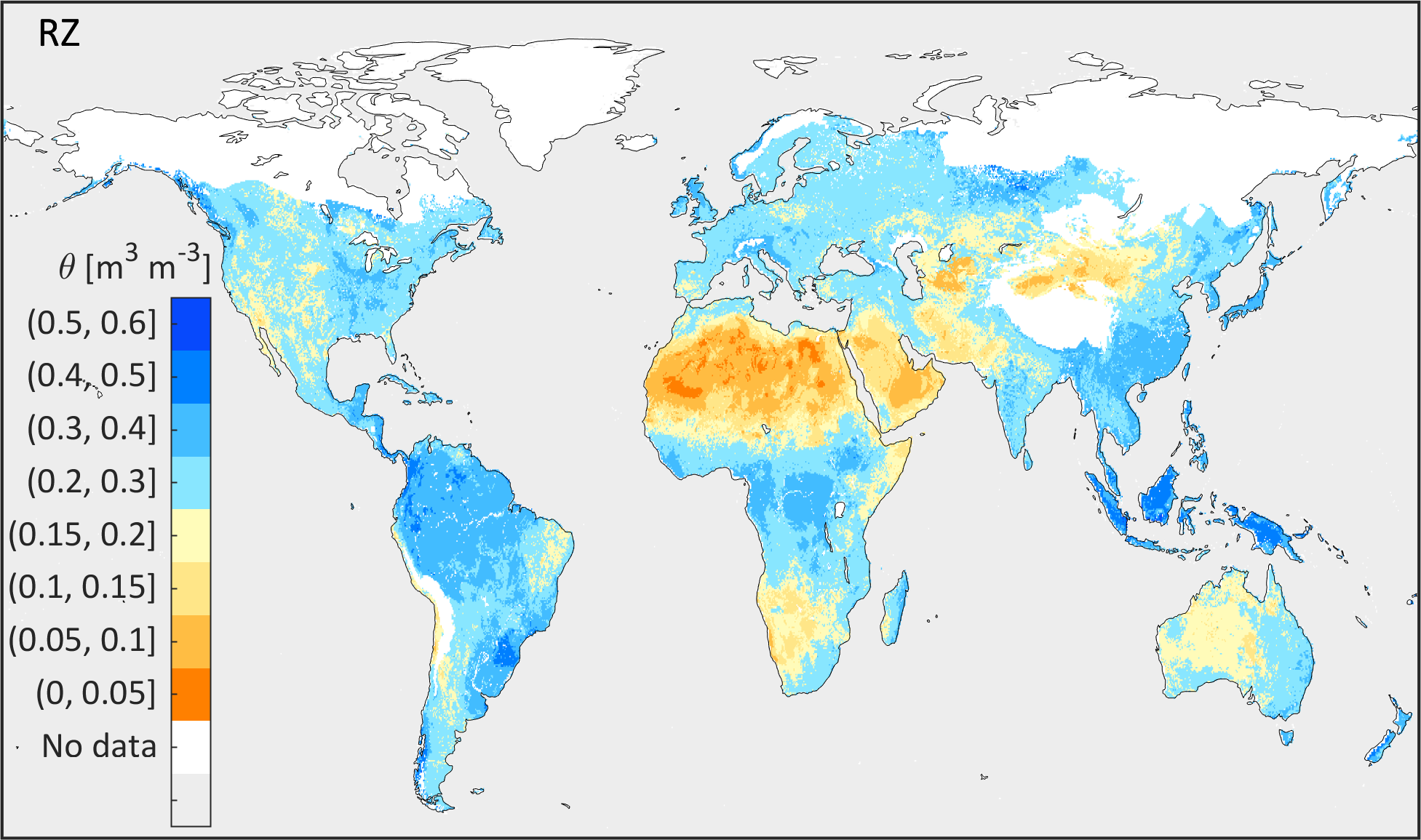


(a)

(b)

**Figure S9**. Geographic distribution of the temporal correlation between the monthly volumetric soil water content $\theta$ in the top soil (TS, 0 to 30 cm) of SOIL-WATERGRIDS and the average value of the ESA/CCI, NOAH/GLDAS, and GLEAM datasets over the period of assessment from 1970 to 2014.


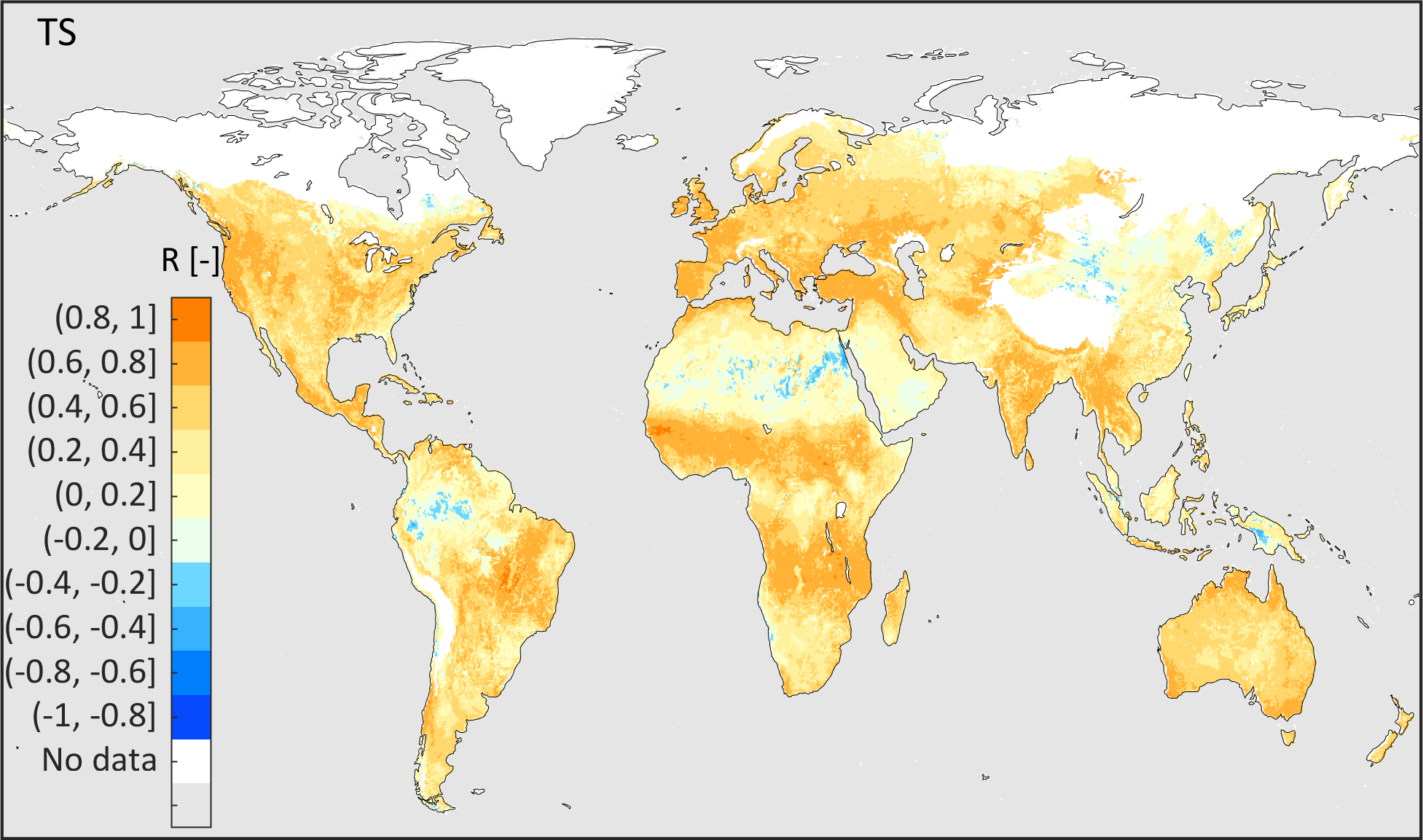

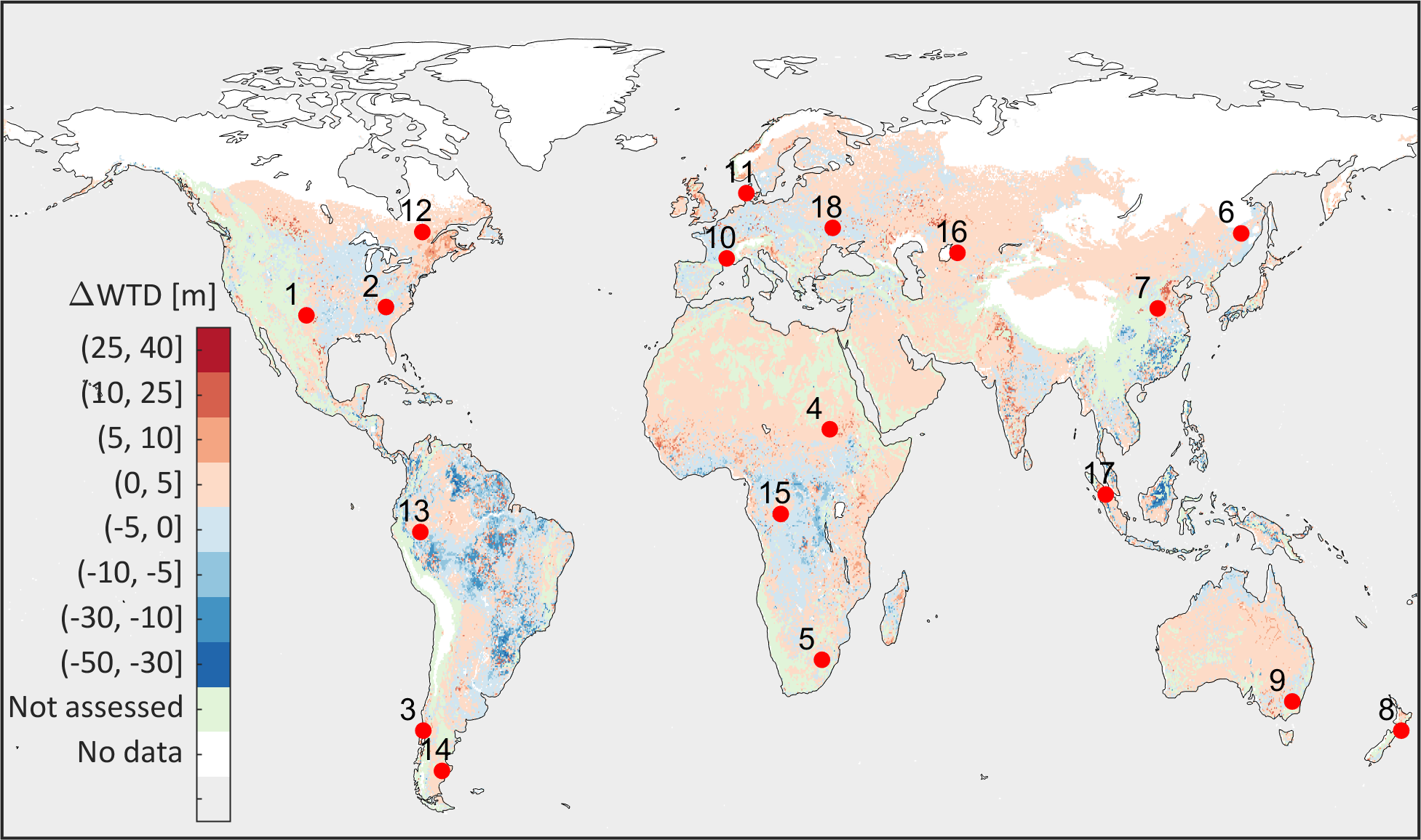


**Figure S11**. Geographic distribution of the anomaly Δ$\mathrm{WTD}$ of the depth of water table (WTD) as compared to data in Fan et al., (2017). An anomaly Δ$\mathrm{WTD}$ > 0 means that the water table is closer to land surface. Point locations correspond to those in Figure 4 of the main paper (Guglielmo et al., 2021).


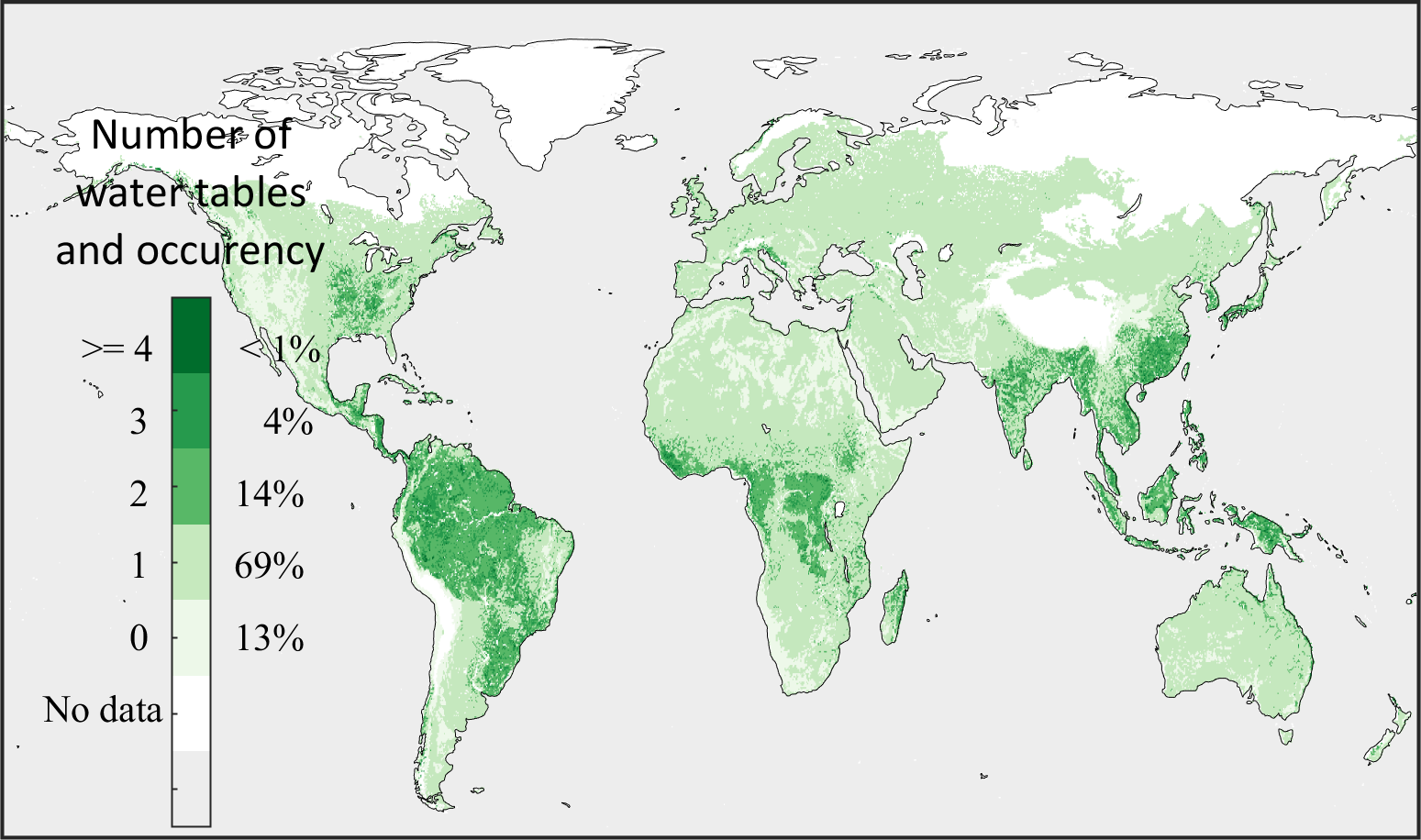

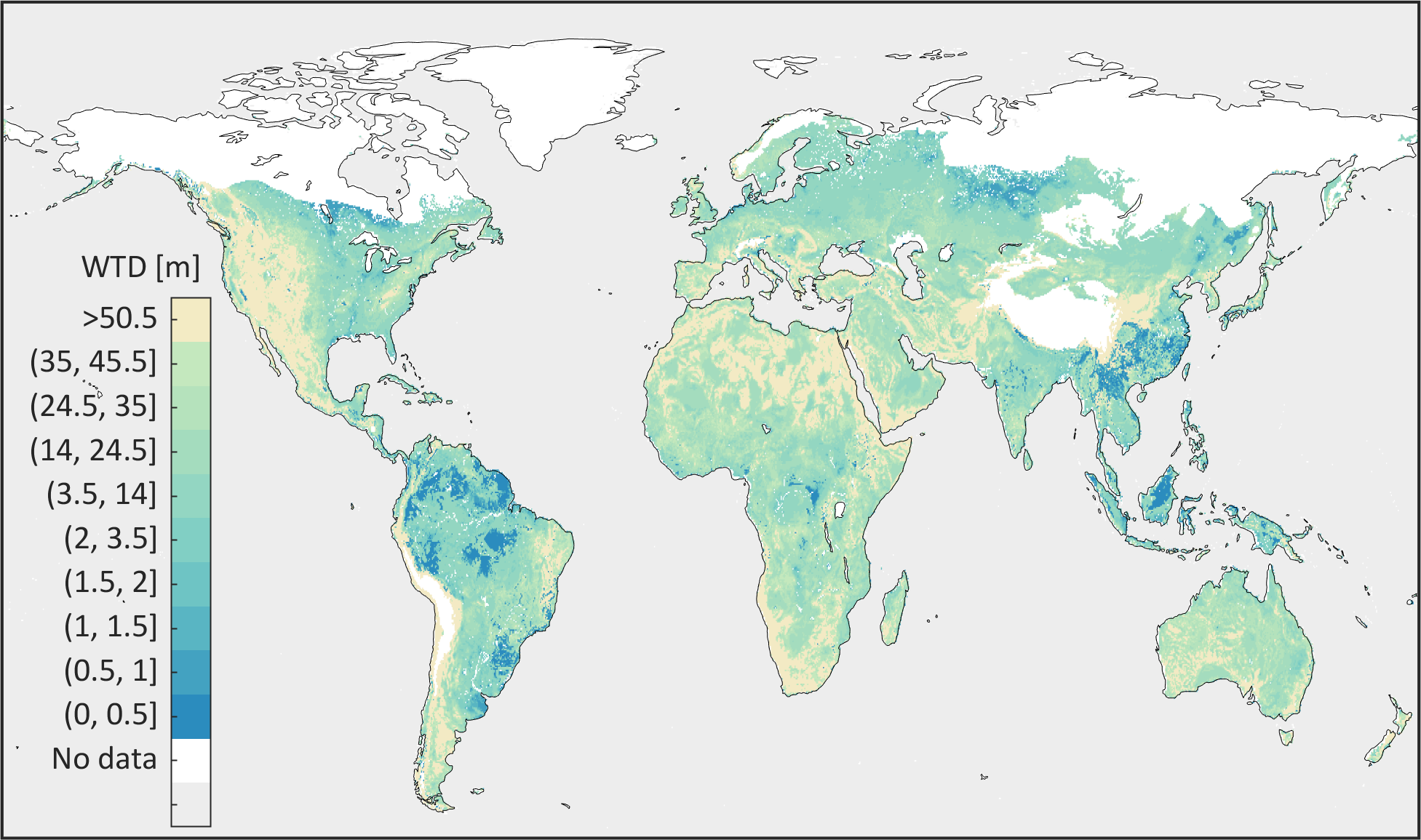


(a)

**Figure S12**. (a) Maximum number of water tables observed during the assessment period from 1970 to 2014. (b) long-term mean depth of the water table closest to data in Fan et al. (2017). The long-term mean is calculated over the assessment period from 1970 to 2014.

(b)

**Figure S14**. Runoff accounting in SOIL-WATERGRIDS (a) excluding and (b) including the water balance as compared to the upper bound represented by the total runoff in GRUNv1 (Ghiggi et al., 2019).


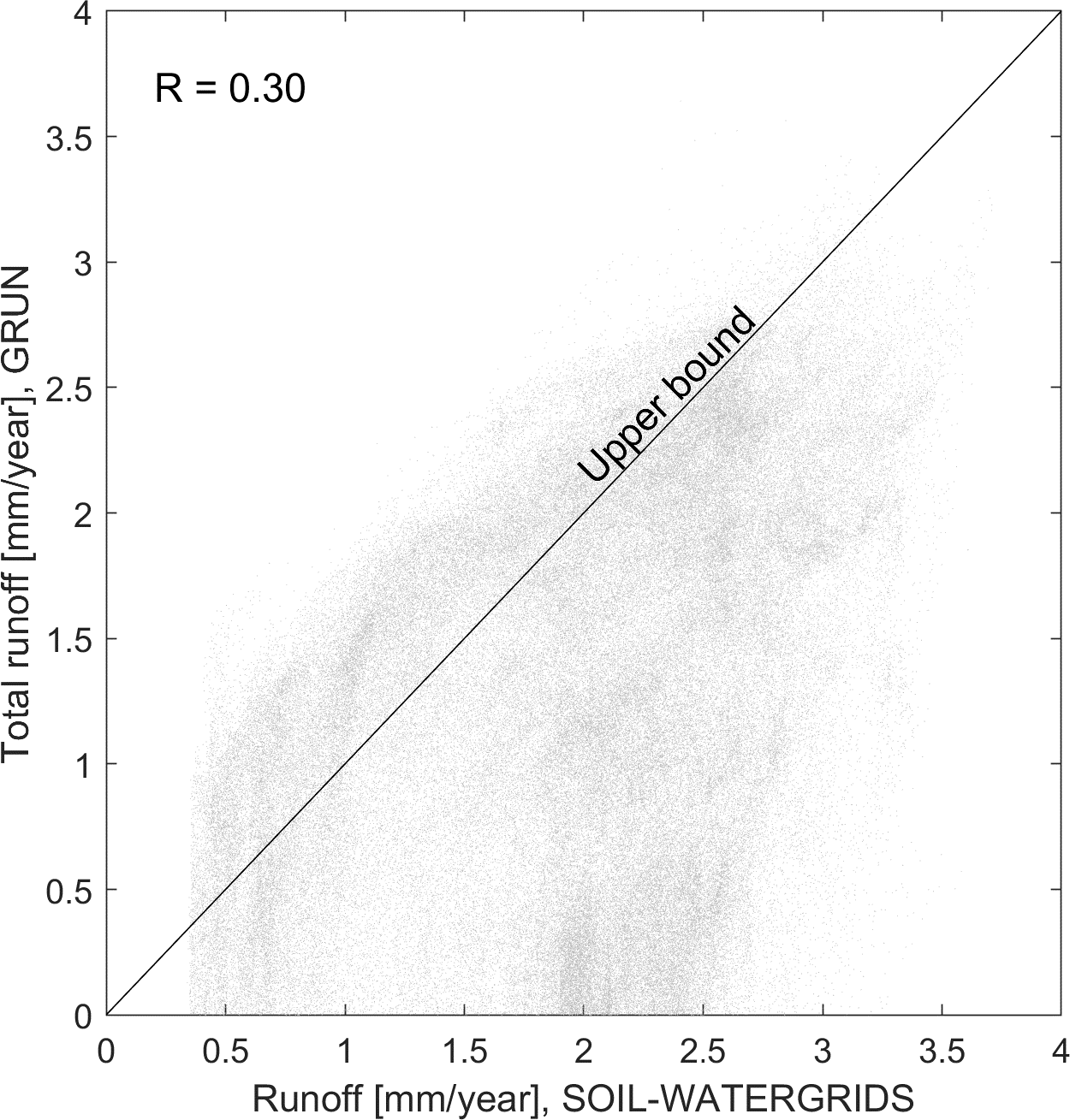

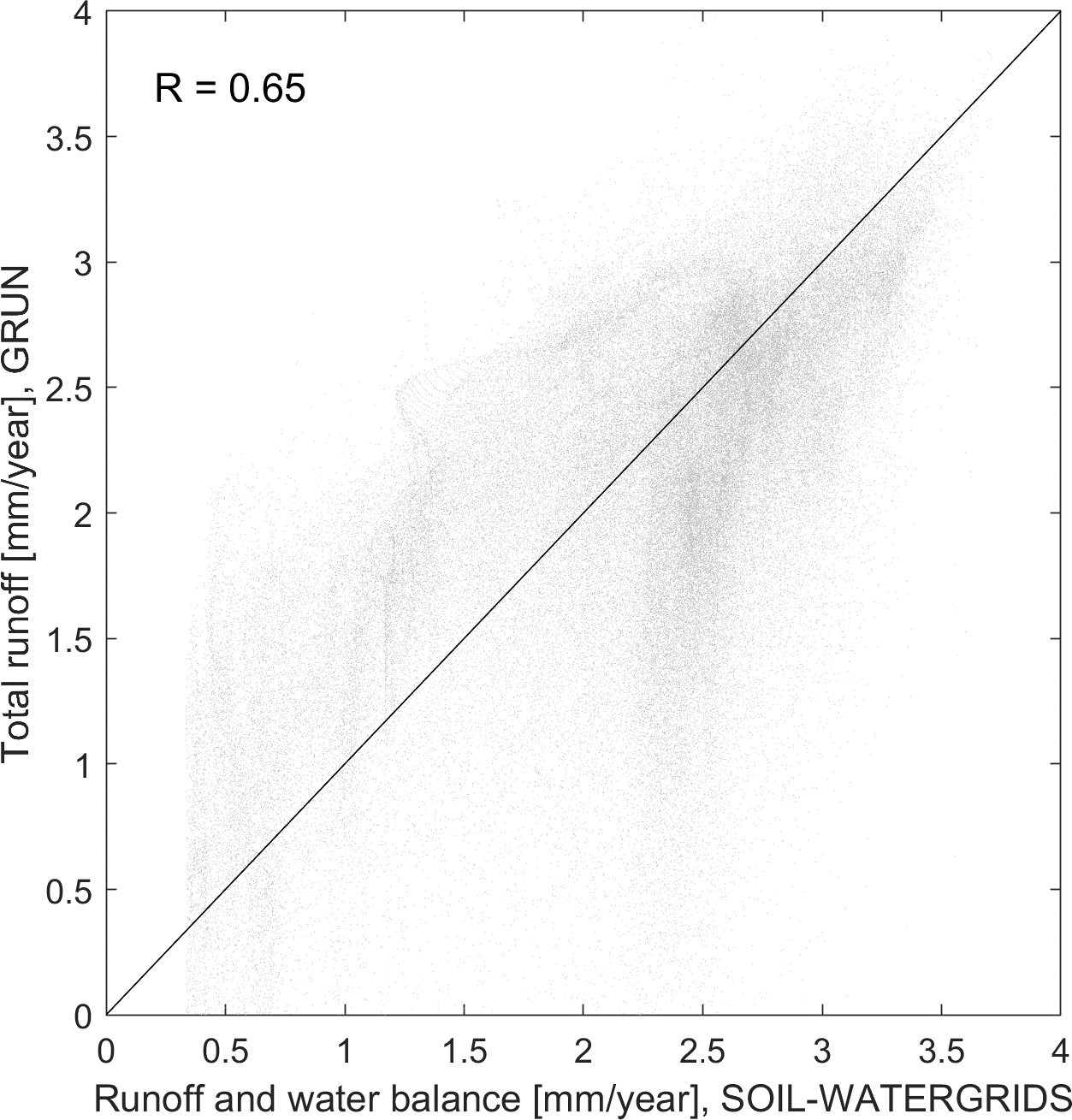


(a) (b)

**Figure S13**. Geographic distribution of ponding occurrence in SOIL-WATERGRIDS and wetlands in SWAMPS (Poulter et al., 2017).


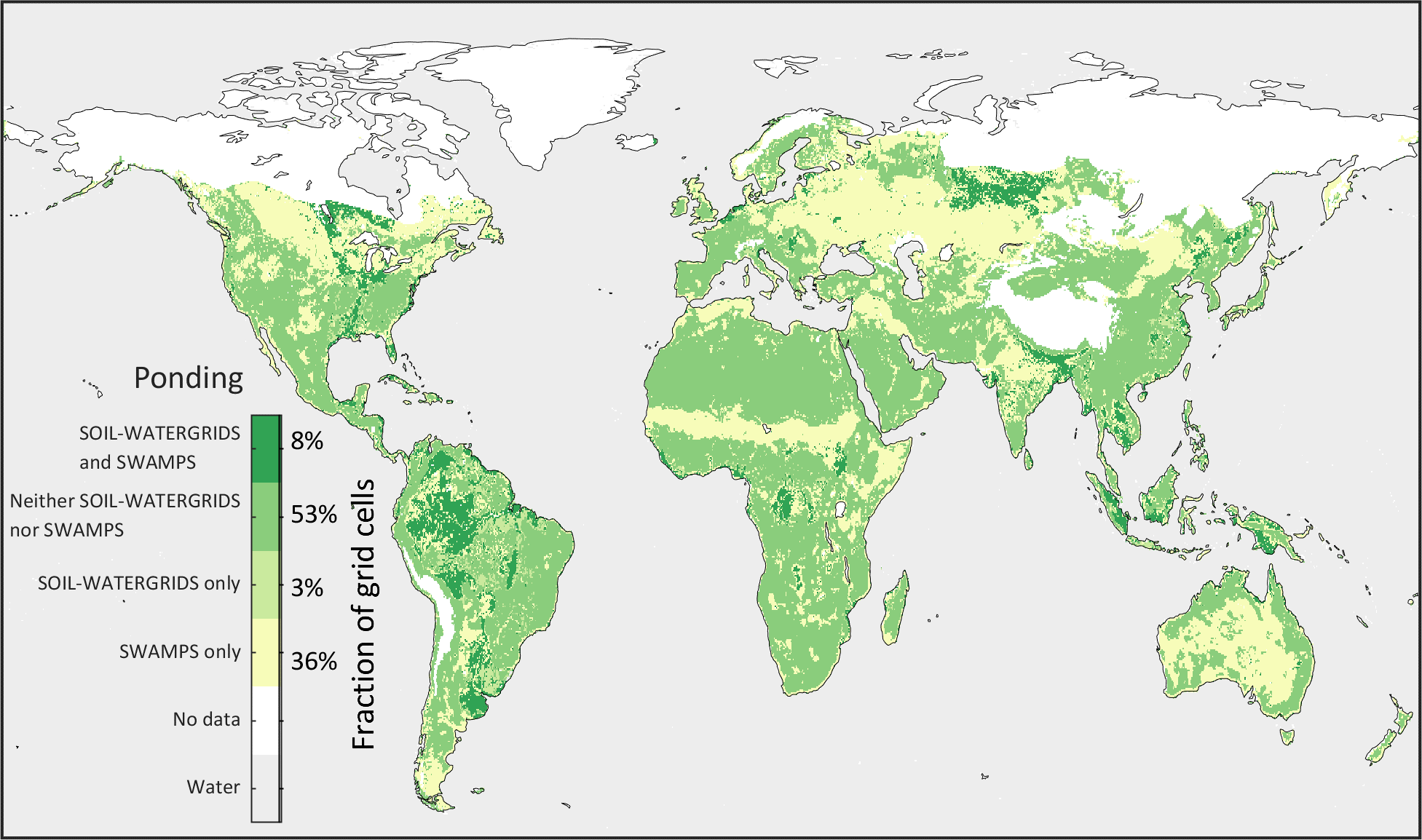

Supplement: Supplementary file 1 — Supplementary Information [file 41597_2021_1032_MOESM1_ESM.docx]
